# Phosphorylation of the auxin signaling transcriptional repressor IAA15 by MPKs is required for the suppression of root development under drought stress in Arabidopsis

**DOI:** 10.1093/nar/gkac798

**Published:** 2022-09-26

**Authors:** Sun Ho Kim, Sunghwa Bahk, Nhan Thi Nguyen, Minh Le Anh Pham, Ulhas Sopanrao Kadam, Jong Chan Hong, Woo Sik Chung

**Affiliations:** Division of Applied Life Science (BK21 Four Program), Plant Molecular Biology and Biotechnology Research Center, Gyeongsang National University, Jinju 52828, Republic of Korea; Division of Applied Life Science (BK21 Four Program), Plant Molecular Biology and Biotechnology Research Center, Gyeongsang National University, Jinju 52828, Republic of Korea; Division of Applied Life Science (BK21 Four Program), Plant Molecular Biology and Biotechnology Research Center, Gyeongsang National University, Jinju 52828, Republic of Korea; Division of Applied Life Science (BK21 Four Program), Plant Molecular Biology and Biotechnology Research Center, Gyeongsang National University, Jinju 52828, Republic of Korea; Division of Applied Life Science (BK21 Four Program), Plant Molecular Biology and Biotechnology Research Center, Gyeongsang National University, Jinju 52828, Republic of Korea; Division of Applied Life Science (BK21 Four Program), Plant Molecular Biology and Biotechnology Research Center, Gyeongsang National University, Jinju 52828, Republic of Korea; Division of Applied Life Science (BK21 Four Program), Plant Molecular Biology and Biotechnology Research Center, Gyeongsang National University, Jinju 52828, Republic of Korea

## Abstract

Since plants are sessile organisms, developmental plasticity in response to environmental stresses is essential for their survival. Upon exposure to drought, lateral root development is suppressed to induce drought tolerance. However, the molecular mechanism by which the development of lateral roots is inhibited by drought is largely unknown. In this study, the auxin signaling repressor IAA15 was identified as a novel substrate of mitogen-activated protein kinases (MPKs) and was shown to suppress lateral root development in response to drought through stabilization by phosphorylation. Both MPK3 and MPK6 directly phosphorylated IAA15 at the Ser-2 and Thr-28 residues. Transgenic plants overexpressing a phospho-mimicking mutant of IAA15 (IAA15^DD^ OX) showed reduced lateral root development due to a higher accumulation of IAA15. In addition, MPK-mediated phosphorylation strongly increased the stability of IAA15 through the inhibition of polyubiquitination. Furthermore, IAA15^DD^ OX plants showed the transcriptional downregulation of two key transcription factors *LBD16* and *LBD29*, responsible for lateral root development. Overall, this study provides the molecular mechanism that explains the significance of the MPK-Aux/IAA module in suppressing lateral root development in response to drought.

## INTRODUCTION

As global warming accelerates, plants are at an increased risk of exposure to many environmental stresses. Drought constitutes a major factor that seriously affects plant growth and development. Roots are the primary plant organs used to detect alterations in the soil status (1) and help recognize drought signals. Alterations in the root system architecture (RSA) in response to drought are considered significant for drought tolerance (2). Such changes in the RSA include the inhibition of lateral root development and the expansion of long primary roots deep into the soil to reach available water (3–5). However, the molecular mechanism underlying the inhibition of lateral root development due to drought is still largely unknown. It is essential to elucidate this mechanism to determine how plants sense drought tolerance and alter lateral root development, which could be used to develop plants with better performance during climate change.

Auxin regulates almost all facets of plant growth and development, including stem elongation, the branching of roots and shoots, embryonic polarity, vascular development, and tropic responses (6–9). Auxin signaling is controlled by the following three core components: TRANSPORT INHIBITOR RESPONSE 1/AUXIN SIGNALING F-BOX PROTEIN (TIR1/AFB) auxin coreceptors, Auxin/INDOLE-3-ACETIC ACID (Aux/IAA) transcriptional repressors, and the AUXIN RESPONSE FACTOR (ARF) transcription factors (10,11). The Aux/IAA proteins repress the transcription of auxin-responsive genes by negatively regulating the transcriptional activity of ARFs (12,13). Upon an auxin stimulus, it promotes interactions between TIR1/AFBs and Aux/IAA proteins, resulting in the degradation of Aux/IAAs via SCF^TIR1/AFB^-E3 ubiquitin ligase-mediated ubiquitination and the release of ARF from transcriptional repression (12,14–16).

The involvement of auxin in lateral root development has been well studied using various auxin-deficient mutants in *Arabidopsis*. For example, analyses of loss-of-function mutants have elucidated that ARF7 and ARF19 positively regulate the transcription of *LBD16* and *LBD29*, which are key transcription factors in lateral root development (17–20). Gain-of-function mutants of Aux/IAA, including *IAA3*/*SHY2*, *IAA14*/*SLR*, *IAA15*, *IAA19*/*MSG2* and *IAA28*, showed severely reduced lateral roots (21–25), suggesting that these Aux/IAAs inhibit the transcriptional activity of specific ARFs required for lateral root development. These studies indicate that several Aux/IAA-ARF modules regulate auxin signaling and control lateral root development.

MPK cascades are evolutionarily conserved in eukaryotes and are involved in various physiological responses (26–29). MPK cascades are also activated in response to stresses and promote stress tolerance in plants (30–33). Moreover, activated MPK cascades lead to the inhibition of auxin signaling, which contributes to stress tolerance. For instance, the activation of MPK cascades by oxidative stress represses the transcription of the auxin-response gene *GH3* (34). Additionally, the MKK7-MPK6 cascade plays a vital role in plant development by regulating polar auxin transport (35). Furthermore, MPK12 was identified as a negative regulator of auxin signaling (36). These observations suggest that MPK cascades play a potential role in the inhibition of auxin signaling under environmental stresses. Hence, studies on the substrates of MPK that inhibit auxin signaling are required to determine the molecular mechanism linking sensing environmental stresses and suppressing plant development.

Recently, we reported a gain-of-function mutant of IAA15, a repressor of auxin signaling, showing defective lateral root development in Arabidopsis (25). Previously, IAA15 was found to be a substrate of MPK using protein microarray technology (37). However, the biological role of IAA15 phosphorylation has not been investigated. In this study, we investigated the physiological function of IAA15 phosphorylation mediated by MPKs. We found that IAA15 can be phosphorylated by both MPK3 and MPK6 *in vitro* and *in vivo*. Interestingly, IAA15^DD^ OX plants showed reduced lateral roots. MPK phosphorylation induces the stabilization of the IAA15 protein by inhibiting polyubiquitination. Furthermore, we observed that the reduced lateral root development in IAA15^DD^ OX plants is caused by the downregulation of *LBD* genes, leading to enhanced tolerance to drought. Our study reveals a significant mechanism of inhibiting auxin signaling by an MPK-IAA15 module, which causes alteration in lateral root development and enhances tolerance to drought.

## MATERIALS AND METHODS

### Plant materials and growth conditions

The *Arabidopsis thaliana* Columbia (Col-0) ecotype and *Nicotiana benthamiana* (tobacco) plants were used in this study. All mutants and transgenic plants used in this study are on the Col-0 background. The synthetic auxin-responsive promoter (*DR5)*-GUS (*DR5*::*GUS*) and NtMEK2^DD^ transgenic plants and *mpk3-1* (SALK_151594) and *mpk6-3* (SALK_127507) mutants were previously described (14,38,39). T-DNA insertion mutant alleles of *iaa15-1* (SAIL_448_B12) and *iaa15-2* (SALK_150265) were obtained from the ABRC (https://abrc.osu.edu).

For surface sterilization, seeds were soaked in 70% EtOH for 1 min, followed by 1/10-diluted commercial bleach (0.4% NaOCl) for 10 min and four washes with sterile distilled water. Surface-sterilized seeds were sown on solid agar plates that consisted of Murashige-Skoog (MS) salts (40), vitamins, and 2.0% sucrose. After stratification for 3 days, the plates were incubated in a growth chamber under a 16 h light/8 h dark photoperiod with a light intensity of ∼120 μmol m^−2^ s^−1^ at 22°C. Ten- to twelve-day-old plants were transplanted into soil and then grown under the same conditions. For the mannitol or NAA treatments, four-day-old plants vertically grown on the surface of MS medium were transferred and vertically grown on MS medium with or without mannitol or auxin. The root growth of the plants was measured by counting the primary root length and lateral root number at 7 d after the transfer.

### Histochemical GUS assays

A GUS assay was performed as previously described (25). To investigate GUS expression in the roots in response to mannitol, *DR5*::*GUS* plants were treated with or without NAA in the presence of mannitol and incubated in 2 mM 5-bromo-4-chloro-3-indolyl-β-d-glucuronic acid (X-gluc) in 50 mM phosphate buffer, pH 7.0, containing 0.5 mM K_3_Fe(CN)_6_ and 0.5 mM K_4_Fe(CN)_6_ for 6 h at 37°C. The tissue was rinsed with 50 mM phosphate buffer, fixed, and cleared overnight in ethanol (100%): acetic acid (9:1, v/v) at room temperature. The samples were imaged under a Nikon SMZ1000 stereoscopic microscope equipped with an OLYMPUS C-5050 ZOOM digital camera.

### Expression of recombinant proteins

Full-length IAA15 and MPKs were amplified by PCR from a cDNA library of Arabidopsis plants using gene-specific primers (Supplementary Table S1). The amplicons were cloned into T-blunt vectors (Solgent, Korea), and their fidelity was verified by sequencing. Another 28 Aux/IAA isoform genes were also amplified by PCR from the cDNA library and cloned into T-blunt vectors (Solgent), and their fidelity was verified by sequencing. To subclone GST-IAA cDNAs, the inserts were excised with *Bam*HI and *Eco*RI and ligated into pGEX-4T-1 vectors (Amersham Biosciences, USA). Double amino acid substitutions (S2A/T28A) in the full-length GST-IAA15 sequence were produced by site-directed mutagenesis using the primers listed in Supplementary Table S1. The mutation was confirmed by sequencing. The GST fusion constructs were transformed into BL21 (DE3) *E*scherichia *coli*, and the GST fusion proteins were expressed and purified using glutathione Sepharose-4B beads (Sigma–Aldrich, USA). To purify the GST- or His-MPKs, the inserts were excised with *Bam*HI and *Sal*I and ligated into pGEX-4T-1 or pQE30 (Qiagen, Germany) vectors. The His-fusion constructs were transformed into *E*. *coli* (M15), and His fusion proteins were expressed and purified using Ni-NTA agarose beads (Qiagen).

### *In vitro* pull-down assays

In total, 2 μg of purified GST and GST-IAA15 were incubated with 10 μl of glutathione-Sepharose 4B beads in pull-down buffer (50 mM Tris–HCl, pH 7.5, 200 mM NaCl, 1% Triton X-100, 0.1 mM EDTA, 0.5 mM DTT, and a protease inhibitor cocktail) for 4 h at 4°C. One microgram of His-MPK3, His-MPK4, or His-MPK6 were added and incubated overnight at 4°C. After washing five times with pull-down buffer, the precipitated beads were collected by brief centrifugation and then eluted by boiling in SDS–PAGE loading buffer. The eluted proteins were separated by SDS-PAGE, transferred onto a PVDF membrane (Bio-Rad, USA), and detected with anti-His antibodies (Abcam, UK).

### Yeast two-hybrid analysis

Full-length IAA15^WT^ was amplified using gene-specific primers (Supplementary Table S1) and cloned into pGAD424 (AD, Clontech) containing the *Leu2* selection marker. MPK3, MPK6, TIR1, ARF7 and ARF19 were amplified using gene-specific primers (Supplementary Table S1) and cloned into pAS2-1 (BD, Clontech) containing the *Trp1* selection marker. The AD and BD plasmids were cotransformed into the yeast strain pJ69-4A (41) and then plated on SD/-Leu/-Trp (-L/W) medium (to select the introduced plasmids). The positive bait−prey interactions of the transformants were tested by monitoring the activation of *HIS3* or *LacZ* reporter genes in growth and *β*-galactosidase assays, respectively.

### Firefly luciferase (LUC) complementation imaging (LCI) assay

The LCI assay was performed as previously described (42); briefly, full-length MPKs and IAA15 were ligated into the pCAMBIA1300-NLuc and pCAMBIA1300-CLuc vectors to produce *35S*::*MPKs*-*NLuc* and *35S*::*CLuc*-*IAA15*, respectively. The resulting constructs were transformed into *Agrobacterium* strain GV3101 (pMP90). *Agrobacterium* cells harboring different constructs were infiltrated into *N. benthamiana* leaves. Three days after the infiltration, the infiltrated leaves were sprayed with luciferin (100 μM, 0.01% Triton X-100) and incubated for 4 h in the dark. LUC activity was detected by a low-light EMCCD apparatus (AndoriXon; Andor, UK).

### *In vitro* and *in-gel* phosphorylation assays

The *in vitro* phosphorylation assay was performed as previously described (32). GST-MPK3 or GST-MPK6 was used to phosphorylate GST (1 μg; negative control), myelin basic protein MBP (0.5 μg; positive control), GST-IAA15^WT^ (2 μg), and GST-IAA15^AA^ (2 μg) in kinase buffer (25 mM Tris–HCl, pH 7.5, 1 mM DTT, 20 mM MgCl_2_, 2 mM MnCl_2_ and 1 μCi [γ-^32^P] ATP) at 30°C for 30 min. The reactions were stopped by the addition of 4× SDS sample buffer, followed by boiling for 5 min. The reaction products were visualized by autoradiography and Coomassie Brilliant Blue (CBB) staining after separation in a 10% SDS–PAGE gel.

The *in-gel* phosphorylation assays were performed as previously described with minor modifications (32). Briefly, the total proteins were extracted from two- to three-week-old plants of Col-0, *mpk3-1* and *mpk6-3* treated with mannitol for the indicated times. Thirty micrograms of total protein were incubated at 60°C for 10 min and then separated by 10% SDS–PAGE on a separating gel containing 0.1 mg/ml MBP (positive control), 0.5 mg/ml purified GST-IAA15^WT^ or GST-IAA15^AA^ as a substrate. After electrophoresis, the gel was washed three times with washing buffer (25 mM Tris–HCl, pH 7.5, 0.5 mM DTT, 0.1 mM Na_3_VO_4_, 5 mM NaF, 0.5 mg/ml bovine serum albumin and 0.1% Triton X-100) to remove the SDS. The proteins were renatured by incubating the gel overnight at 4°C in renaturing buffer (25 mM Tris–HCl, pH 7.5, 1 mM DTT, 0.1 mM Na_3_VO_4_, and 5 mM NaF), with three buffer changes. The gel was equilibrated in reaction buffer (25 mM Tris–HCl, pH 7.5, 2 mM EGTA, 12 mM MgCl_2_, 1 mM DTT and 0.1 mM Na_3_VO_4_) at 30°C for 30 min. The kinase reaction was carried out by incubating the gel at 30°C for 1.5 h in 20 ml of reaction buffer consisting of 0.5 μM ATP and 50 μCi [γ-32P] ATP. The reaction was stopped by transferring the gel to a stop solution (5% trichloroacetic acid and 1% disodium pyrophosphate). The gels were washed with the stop solution for 5 h at room temperature, with four changes of the solution; then the gels were dried on 3M paper. MPK3 and MPK6 kinase activity was detected with autoradiography.

### Mass spectrometric analysis of phosphopeptides using TiO_2_ microcolumns

GST-IAA15 was treated with GST-MPK6 as described above for the *in vitro* phosphorylation assay. The protein bands were excised after separation on SDS-PAGE gels and *in-gel* digestion with modified trypsin (Promega, USA) (43). The digested peptides were subsequently dissolved in loading buffer (80% acetonitrile and 5% trifluoroacetic acid) and passed through a TiO_2_ microcolumn. The eluted phosphopeptides with NH_4_OH (pH 10.5) were added to a Poros Oligo R3 column (Applied Biosystems, USA) and then eluted from the column using 2,5-dihydroxybenzoic acid (DHB; Fluka, USA) solution (20 mg/ml DHB, 50% acetonitrile, 0.1% trifluoroacetic acid, and 1% orthophosphoric acid). A MALDI-MS analysis was performed using a Voyager-DE STR mass spectrometer (PerSeptive Biosystems, Inc., USA). The mass spectra were obtained with the instrument in the reflectron/delayed extraction mode. Monoisotopic peptide masses were analyzed using MoverZ software (www.proteometric.com).

### *In vivo* phosphorylation assay

IAA15 protein phosphorylation *in vivo* was detected by a mobility shift assay using a Phos-tag reagent (NARD Institute, Japan). The total proteins were extracted from 2-week-old IAA15^WT^ OX plants treated with mannitol and separated on a 10% SDS–PAGE gel containing 100 μM phos-tag and 200 μM ZnCl_2_. After electrophoresis, the gel was incubated in transfer buffer containing 10 mM EDTA three times, washed with transfer buffer (50 mM Tris, 40 mM glycine) for 10 min, and then transferred onto a PVDF membrane (Bio-Rad). The IAA15 proteins were detected with anti-Flag antibodies (1:5000; Sigma–Aldrich).

### Construction of transgenic plants

To generate the *35S*::*3XFLAG*-*IAA15^WT^*, *35S*::*3XFLAG*-*IAA15^AA^* and *35S*::*3XFLAG*-*IAA15^DD^* constructs, amino acid substitutions (S2A/T28A or S2D/T28D) in full-length IAA15 were produced by site-directed mutagenesis using the primers listed in Supplementary Table S1. *IAA15^WT^* and its mutants were subcloned into the *Bam*HI and *Spe*I sites of pBlueScript II KS (+) (Stratagene, USA) containing *3XFLAG*. The *3XFLAG*-*IAA15^WT^*, *3XFLAG*-*IAA15^AA^* and *3XFLAG*-*IAA15^DD^* constructs were cloned into pCAMBIA 1300 binary vectors (Abcam) containing the *35S* promoter. All constructs were confirmed by sequencing and transformed into Col-0 or *DR5*::*GUS* plants using *Agrobacterium* strain GV3101 (pMP90) by the floral-dip method (44). The transformants were selected on MS media containing the proper antibiotics. T_3_ homozygous progeny of the transgenic plants expressing high levels of IAA15 were used in all experiments.

To generate *IAA15_pro_*::*3XFLAG*-*IAA15^WT^*-*GUS*, *IAA15^WT^* cDNA was cloned into the *Bam*HI and *Spe*I sites of pBlueScript II KS (+) containing *3XFLAG*-*GUS*. A 2.4 kb upstream region of the ATG start codon in the *IAA15* genome was cloned into the *Hind*III and *Sal*I sites of pBlueScript II KS (+) containing *3XFLAG*-*IAA15^WT^*-*GUS*. *IAA15_pro_*::*3XFLAG*-*IAA15^WT^*-*GUS* was cloned into the HindIII and *Sac*I sites of the pPZP211 binary vector (45). The construct was confirmed by sequencing and transformed into Col-0 plants. The transformants were selected on MS media containing the proper antibiotics. T_3_ homozygous progeny of transgenic plants expressing high levels of IAA15 were used in all experiments.

To generate double transgenic plants coexpressing FLAG-NtMEK2^DD^/hemagglutinin (HA)-IAA15^WT^, *IAA15^WT^* was cloned into a pMLBart vector containing the HA epitope under the control of the *35S* promoter. The construct was then transformed into Flag-NtMEK2^DD^ OX plants controlled by a dexamethasone (DEX)-inducible promoter. The double transformants were selected on MS medium containing the proper antibiotics. T_3_ homozygous progeny of transgenic plants expressing high levels of IAA15 were used in all experiments.

### Protein extraction and immunoblot analysis

The total plant proteins were extracted from 2-week-old plants treated with mock, MG132, or mannitol in protein extraction buffer (50 mM Tris–HCl, pH 7.5, 5 mM EDTA, 5 mM EGTA, 1 mM Na_3_VO_4_, 25 mM NaF, 50 mM glycerophosphate, 2 mM DTT, 2 mM PMSF, 5% glycerol, 1% Triton X-100, and protease inhibitor cocktail), and separated by SDS–PAGE. The IAA15 proteins were detected with anti-FLAG antibodies (1:5000; Sigma–Aldrich).

To detect ubiquitinated IAA15, the total proteins were extracted from 2-week-old plants of IAA15^WT^ and IAA15^DD^ OX treated with or without NAA plus MG132 for 24 h and then immunoprecipitated using anti-Flag antibodies coupled to agarose beads (Sigma–Aldrich) for 4 h at 4°C. The immunoprecipitated proteins were detected by immunoblotting with anti-ubiquitin antibodies (Santa Cruz Biotechnology, USA).

For the *in vivo* turnover assay of IAA15 proteins, the total proteins were extracted from 2-week-old plants of IAA15^WT^ and IAA15^DD^ OX, which were pretreated with 10 μM MG132 or 200 mM mannitol for 20 h, treated with or without 10 μM NAA in the presence of 1 mM cycloheximide (CHX) for 4 h and separated by SDS–PAGE. The IAA15 proteins were detected by immunoblotting with anti-Flag (1:5,000; Sigma–Aldrich).

To estimate the activities of MPK3 and MPK6, the total proteins were extracted from 2-week-old NtMEK2^DD^ OX/IAA15^WT^ OX plants treated with 10 μM MG132 and 50 μM DEX and separated by SDS–PAGE. The activities of MPK3 and MPK6 were detected by immunoblotting with anti-p44/42 antibodies (1:10,000; Cell Signaling Technology, USA).

### Cell-free protein degradation assay

The cell-free protein degradation assay of IAA15 was performed as previously described (46,47) with minor modifications. The total proteins were extracted from 2-week-old plants of Col-0, *mpk3*, and *mpk6* in degradation buffer (50 mM Tris–HCl, pH 8.0, 500 mM sucrose, 1 mM MgCl_2_, 10 mM EDTA, pH8.0 and 5 mM DTT). Equal amounts of proteins from Col-0, *mpk3*, and *mpk6* plants were incubated with recombinant GST-IAA15 protein at 25°C for different durations. The IAA15 protein was detected by immunoblotting with anti-GST antibodies (Abm, USA).

### DNA, RNA extraction, and qPCR analysis

Genomic DNA was isolated using an Exogene GeneAll plant SV mini kit (GeneAll Biotechnology, South Korea) according to the manufacturers’ instructions. The total RNA was extracted from 2-week-old plants grown on MS medium using an RNA purification kit (Macherey-Nagel, Germany). Five micrograms of total RNA were reverse transcribed using SuperScript II RNase-Reverse Transcriptase (Invitrogen, USA). qPCR analyses were performed on a CFX384 Real-Time System (Bio-Rad, USA) using iQ SYBR^®^ Green Supermix (Bio-Rad) with gene-specific primers (Supplementary Table S2).

### Chromatin immunoprecipitation (ChIP) assay

For the ChIP assay, 500 mg plants of Col-0, IAA15^WT^ OX, IAA15^AA^ OX, and IAA15^DD^ OX were harvested in PBS and fixed with formaldehyde. The ChIP assays were performed using anti-Flag antibodies (1:3000; Sigma–Aldrich) as previously described (48,49). The qPCR analyses were performed on a CFX384 Real-Time System using iQ SYBR^®^ Green Supermix with gene-specific primers (Supplementary Table S2).

## RESULTS

### Drought inhibits lateral root development via the suppression of auxin signaling by MPK cascades

**Figure 1.**
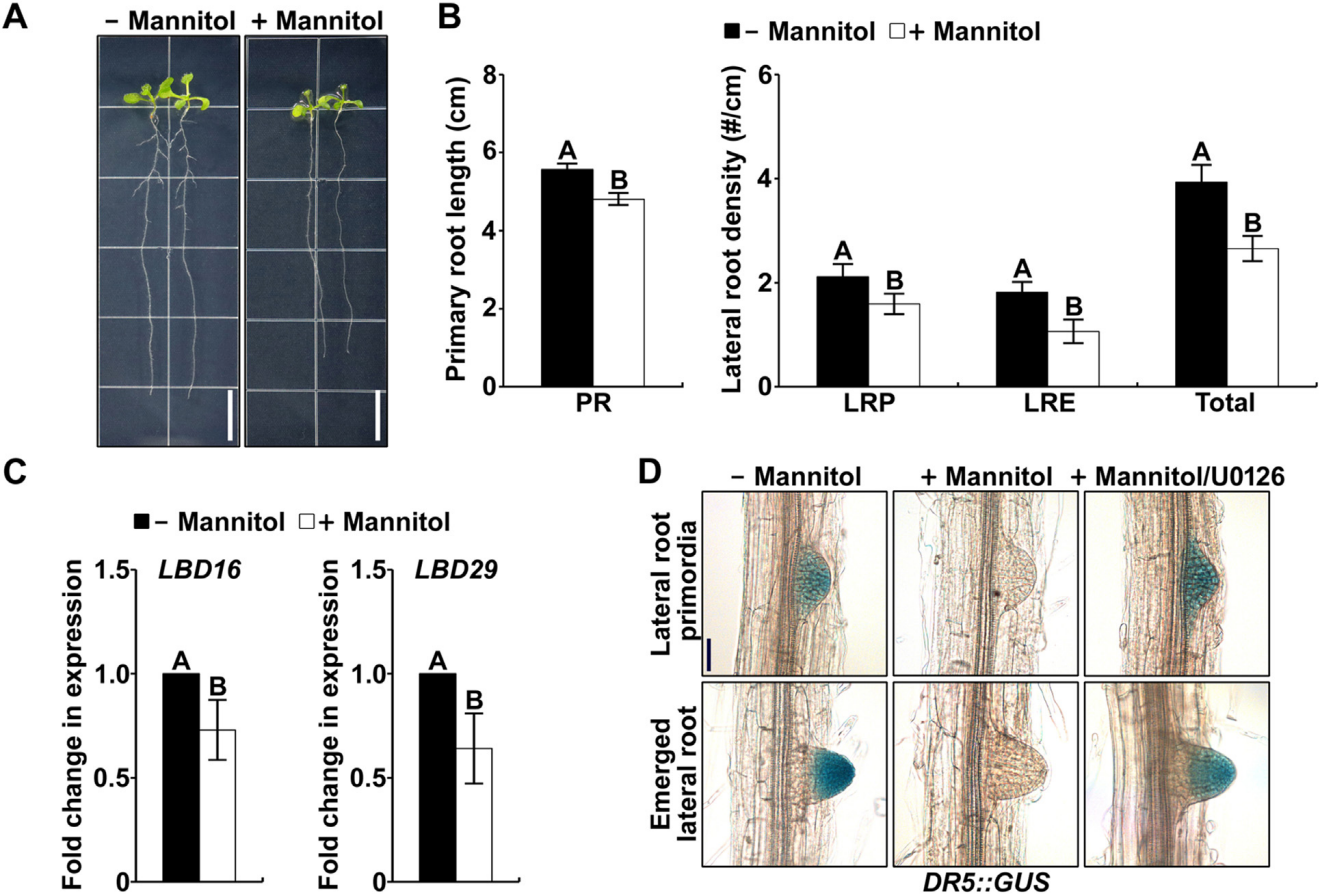
Drought inhibits lateral root development by inhibiting auxin signaling. (**A**) Root morphologies of Col-0 plants grown vertically on MS plates containing with or without 75 mM mannitol. Four-day-old plants grown on MS medium were transferred to the plates shown. The plants were photographed at 7 d after the transfer. The scale bar represents 1 cm. (**B**) Primary root (PR) length and density of lateral root primordia (LRP), emerged lateral roots (LRE), and total lateral roots (LRP + LRE) of Col-0 plants grown vertically on MS plates as shown in Supplementary Figure S1. The bars indicate the mean ± S.D. (*n* = 20). Different letters represent significant differences (*P* < 0.001). (**C**) Transcriptional inhibition of *LBD* genes by treatment with mannitol. Transcript levels of *LBD16* and *LBD29* were measured by qPCR using gene-specific primers. The bars indicate the mean ± S.D. (*n* = 3). Different letters represent significant differences determined with Student's *t*-test (*P* < 0.01). (**D**) Mannitol inhibits auxin signaling through MPK cascade. *DR5*::*GUS* plants were treated with 200 mM mannitol alone or in combination with 10 μM U0126 for 6 h. GUS activities were detected in the lateral root primordia and emerged lateral root. The scale bars represent 50 μm.

It has been reported that lateral root development is inhibited due to water deficit, which increases the tolerance of plants to drought (5,50). To investigate whether drought confers a negative effect on auxin-mediated lateral root development, we treated Col-0 plants with mannitol, which was used to create an artificial drought effect on growth media. As a result, the primary root length and the lateral root density (primordia and emerged) were reduced in response to mannitol (Figure 1A and B; Supplementary Figure S1), confirming that drought inhibits lateral root development. Then, to examine whether auxin signaling in the roots is suppressed by mannitol, we measured the expression of representative auxin-responsive genes encoding key transcription factors in lateral root development, *LBD16* and *LBD29*. The transcription of *LBD16* and *LBD29* genes was obviously decreased by the mannitol treatment (Figure 1C). In addition, we analyzed the auxin response by measuring GUS activity in *DR5*::*GUS* plants. As expected, high GUS activity was detected in the lateral root primordia and emerged lateral roots under normal conditions (Figure 1D). However, the GUS activity in the lateral roots was obviously decreased upon the treatment with mannitol (Figure 1D). These results indicate that drought may suppress lateral root development by inhibiting auxin signaling. However, to date, the signaling pathway involved in the inhibitory effect of drought on auxin signaling has not been investigated. Since auxin signaling is inhibited by the activation of a stress-responsive MPK cascade (34–36), we investigated whether it was also involved in the inhibitory effect of drought. For that purpose, we treated *DR5*::*GUS* seedlings with an MKK inhibitor, U0126. We found that the highly suppressed GUS activity by mannitol was restored upon cotreatment with U0126 (Figure 1D), suggesting that an MPK cascade is involved in the inhibition of auxin signaling in response to drought.

### IAA15 is a potential substrate of drought-responsive MPKs

Since seven Aux/IAA proteins (IAA1, IAA5, IAA8, IAA11, IAA13, IAA15 and IAA31) have been identified as potential substrates of MPKs based on a protein microarray analysis (37), to further validate whether stress-responsive MPKs directly phosphorylate Aux/IAA proteins, we performed *in vitro* phosphorylation assays using recombinant glutathione S-transferase (GST)-fused Arabidopsis IAA proteins and GST-fused MPK3. We found that in total, eleven (IAA4, IAA7, IAA8, IAA12, IAA14, IAA15, IAA18, IAA19, IAA28, IAA29 and IAA34) of the 29 tested recombinant Aux/IAA proteins were phosphorylated by MPK3 (Supplementary Figure S2). In particular, IAA4, IAA7, IAA8, IAA12, IAA14, IAA15, IAA18 and IAA34 were strongly phosphorylated (Supplementary Figure S2), suggesting that many Aux/IAAs are potential substrates of MPKs.

Among these strongly phosphorylated IAAs, IAA14 and IAA15 were previously reported to be negatively involved in lateral root development through gain-of-function mutants (23,25). In addition, it has been reported that the transcripts of *IAA14* and *IAA15* were highly detected in developing lateral roots (23,25). However, their protein levels have not been studied. In this study, to evaluate whether the IAA15 protein is detected in the lateral roots, we constructed transgenic plants expressing the *3XFLAG*-*IAA15^WT^*-*GUS* under the control of the *IAA15* promoter (*IAA15_pro_*::*3XFLAG*-*IAA15^WT^*-*GUS*). As shown in Supplementary Figure S3, IAA15^WT^-GUS activity was not detected in the lateral roots of the transgenic plants (mock) but was highly detected following the treatment with the proteasome inhibitor MG132, indicating that IAA15 is unstable under normal conditions. Interestingly, IAA15^WT^-GUS activity was also highly detected after the treatment with mannitol, suggesting that IAA15 can be stabilized in response to drought in the lateral roots.

### MPK3 and MPK6 phosphorylate IAA15 *in vitro* and *in vivo*

**Figure 2.**
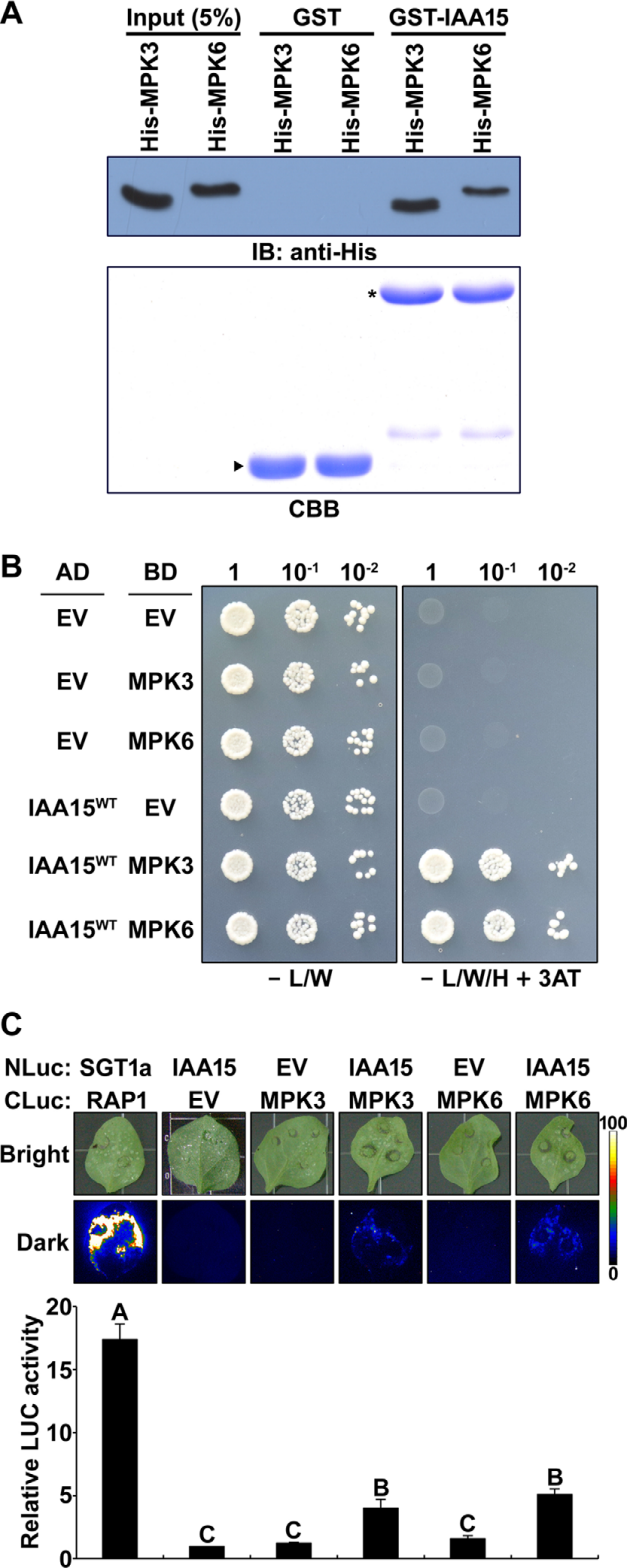
IAA15 interacts with MPK3 and MPK6. (**A**) *In vitro* pull-down assay showing the interaction between IAA15 and MPKs. The interactions between GST-fused IAA15 and His-fused MPK3 and MPK6 were tested. GST-fused proteins were precipitated using Glutathione-Sepharose 4B resin. His-fused MPKs were analyzed by immunoblotting using anti-His antibodies (upper panel). The SDS-PAGE gel was stained by Coomassie Brilliant Blue (CBB) staining (lower panel). Arrowheads and stars indicate GST and GST-IAA15, respectively. (**B**) Yeast two-hybrid analysis showing the interaction of IAA15 with MPK3 or MPK6. The indicated AD and BD plasmids were co-transformed into yeast strain pJ69-4A. Serially diluted transformants were grown on selective SD medium lacking Leu and Trp (-L/W) (control) and on SD medium lacking Leu, Trp, and His (-L/W/H) supplemented with 20 mM 3-amino-1,2,4-triazole (3-AT) to demonstrate activation of the *HIS3* reporter gene. (**C**) Luciferase (LUC) complementation imaging (LCI) assay showing the interaction of IAA15 with MPK3 or MPK6. The upper panel shows the bright field images (bright) and luminescence (dark) of *N*. *benthamiana* leaves co-infiltrated with strains containing the indicated combinations of NLuc- and CLuc-fusion constructs. The *SGT1a*-*NLuc*/*CLuc*-*RAR1* combination was used as a positive control. EV refers to empty vectors of *NLuc* and *CLuc*. Leaves were photographed at 3 days after infiltration. The lower panel shows the quantification of LUC activity as shown in the upper panel. The luminescence intensities were measured relative to leaves infiltrated with the *IAA15*-*NLuc*/*CLuc* (EV) combination. The bars indicate the mean ± S.D. (*n* = 3). Different letters represent significant differences (*P* < 0.01).

To explore whether IAA15 is a substrate of MPKs, we first examined the interaction of IAA15 with MPK3, MPK4, and MPK6 using *in vitro* pull-down and yeast two-hybrid assays. The pull-down assay results showed that GST-IAA15 pulled down all tested MPKs, but GST alone did not pull down any MPKs (Figure 2A; Supplementary Figure S4A). However, the yeast two-hybrid-based analysis indicated that IAA15 interacts with MPK3 and MPK6 but not with MPK4 (Figure 2B; Supplementary Figure S4B). To confirm the interaction between IAA15 and MPKs *in planta*, we performed a luciferase (LUC) complementation imaging (LCI) assay in *Nicotiana benthamiana* leaves. LUC activity was detected in leaves coexpressing IAA15 and MPK3 or MPK6 at levels approximately 4- to 5-fold higher than those in the control leaves coinfiltrated with IAA15 and an empty vector (EV) (Figure 2C), while LUC activity in the leaves coexpressing IAA15 and MPK4 was similarly detected in the leaves coexpressing EV and MPK4 (Supplementary Figure S4C). These results indicate that IAA15 interacts with MPK3 and MPK6 *in planta*.

**Figure 3.**
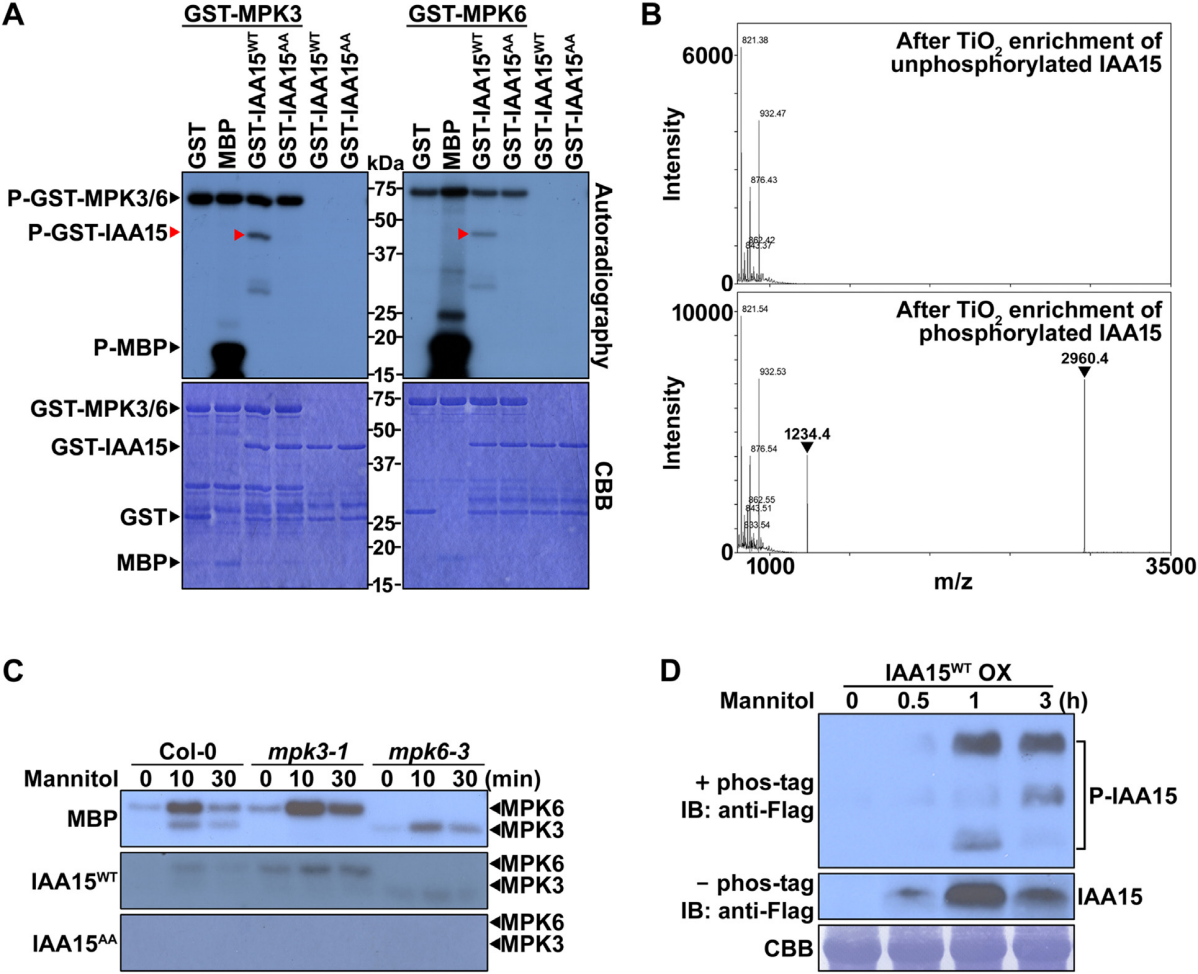
IAA15 is phosphorylated by MPK3 and MPK6. (**A**) *In vitro* phosphorylation of IAA15^WT^ and IAA15^S2A/T28A^ (IAA15^AA^) by recombinant MPK3 and MPK6. Recombinant proteins were separated by 10% SDS-PAGE after incubation in protein kinase buffer containing [γ-^32^P] ATP. Phosphorylated IAA15 was detected by autoradiography after gel electrophoresis (upper panel). Recombinant proteins were detected by CBB staining (lower panel). MBP and GST were used as positive and negative control substrates, respectively. (**B**) Identification of phosphorylation sites of IAA15 by MPK6 using mass spectrometry. Shown are the peptide mass fingerprints of unphosphorylated and MPK6-phosphorylated GST-IAA15 with TiO_2_ purification after trypsin digestion. Two phospho-peptide peaks (arrowheads) were detected from phosphorylated GST-IAA15. (**C**) *In-gel* kinase assay of MPK3 and MPK6 after mannitol treatment. Total proteins were extracted from 2-week-old plants of Col-0, *mpk3-1*, and *mpk6-3* treated with 200 mM mannitol for the indicated times to activate MPK activity. Shown are autoradiographs of SDS-polyacrylamide gels containing MBP, GST-IAA15^WT^ or GST-IAA15^AA^ as a substrate, depicting signals from an *in*-*gel* kinase assay on separated total proteins from extracts of treated plants. The expected positions of MPK3 and MPK6 on the gels are indicated. (**D**) Phos-tag mobility shift assay showing *in planta* phosphorylation of IAA15 in response to mannitol. IAA15^WT^ OX plants were treated with 200 mM mannitol for the indicated times. Protein extracts from IAA15^WT^ OX plants treated with 200 mM mannitol were separated in an SDS gel embedded with phos-tag reagent (upper panel) or without phos-tag reagent (middle panel). IAA15 protein was then detected by immunoblotting (IB) with anti-Flag antibodies. The Rubisco band stained with CBB is shown to verify similar amounts of protein loading (lower panel).

Since IAA15 was phosphorylated by MPK3 (Supplementary Figure S2), we also examined whether IAA15 was phosphorylated by MPK6 via an *in vitro* phosphorylation assay using [γ-^32^P]-labeled ATP with purified recombinant GST-IAA15^WT^ and GST-MPKs. After separating the proteins by SDS–PAGE, we detected the phosphorylated proteins on the gel by autoradiography and visualized the total proteins with CBB staining. As shown, MPK3 and MPK6 phosphorylated myelin basic protein (MBP) and GST-IAA15^WT^ (Figure 3A), but MPK4 did not phosphorylate GST-IAA15^WT^ (Supplementary Figure S4D). No phosphorylation band corresponding to GST was observed.

As MPKs usually phosphorylate the Ser (S) and Thr (T) residues in S/T-P motifs, the following two potential MPK phosphorylation sites were predicted in IAA15: Ser-2 and Thr-28. To identify the MPK phosphorylation sites on IAA15, we performed mass spectrometry after selectively enriching phosphopeptides by TiO_2_ chromatography. Initially, a kinase reaction was performed with GST-IAA15^WT^ in the absence and presence of GST-MPK6. Following separation by SDS–PAGE, we excised the requisite bands corresponding to GST-IAA15^WT^, digested them with trypsin, and subjected the resulting peptides to mass spectrometry. After TiO_2_ chromatography, the following two phosphopeptides were identified containing two predicted phosphorylation sites of MPK6: GSMS_2_PEEYVR (Ser-2) and VWPDSGDLGGTELTLALPGT_28_PTNASEGPK (Thr-28) (Figure 3B; Supplementary Table S3). Therefore, the Ser-2 and Thr-28 residues of IAA15 were identified as two MPK6 phosphorylation sites. To confirm these phosphorylation sites on IAA15, we performed *in vitro* phosphorylation assays after mutating the two residues to Ala (A) (IAA15^S2A/T28A^, designated IAA15^AA^). As a result, IAA15^AA^ was no longer phosphorylated by MPK3 or MPK6 (Figure 3A), indicating that the two residues are the phosphorylation sites of IAA15 for these two MPKs.

To test whether native MPK3 and MPK6 phosphorylate IAA15, we performed an *in-gel* kinase assay using gels embedded with purified GST-IAA15^WT^, GST-IAA15^AA^, and MBP (positive control) and fractionated cell-free extracts prepared from 3-week-old Col-0, *mpk3* and *mpk6* plants treated with mannitol for a short duration because MPKs are known to be rapidly and transiently activated (51–53). Radiolabeled phospho-IAA15 bands at the expected location of MPK3 (∼44 kDa) and MPK6 (∼46 kDa) were detected in the GST-IAA15^WT^-embedded gel containing resolved extracts of Col-0, *mpk3*, and *mpk6* plants (Figure 3C). A similar pattern of phosphorylation was observed in the gel embedded with MBP, but no bands were observed in the gel embedded with GST-IAA15^AA^ (Figure 3C). These results indicate that plant MPK3 and MPK6 function in the phosphorylation of IAA15.

Furthermore, we examined the phosphorylation of IAA15 *in planta* by performing a phos-tag mobility shift assay. A transgenic plant overexpressing *3XFLAG*-*IAA15^WT^* (IAA15^WT^ OX) was constructed and treated with mannitol to activate endogenous MPKs for a relatively long time compared to *in-gel* kinase assay because more time may be necessary for the stabilization of IAA15 proteins after the phosphorylation of MPKs in response to stresses. The IAA15 protein was detected by immunoblotting with anti-Flag antibodies in the gels embedded with or without the phos-tag. As a result, we found that the protein level of IAA15 started to increase 0.5 h after the treatment with mannitol (middle panel, Figure 3D). By increasing the IAA15 protein level, three different shifted bands (P-IAA15) were detected in the gel embedded with phos-tag (upper panel, Figure 3D), indicating that IAA15 can be phosphorylated by mannitol-responsive MPKs as diverse phosphorylated forms. These results demonstrate that IAA15 is phosphorylated in response to mannitol *in planta*, which may contribute to the stabilization of IAA15. Therefore, we suspected that the phosphorylation of IAA15 by drought-responsive MPKs may be involved in the inhibition of lateral root development.

### IAA15 is involved in the suppression of lateral root development under drought

**Figure 4.**
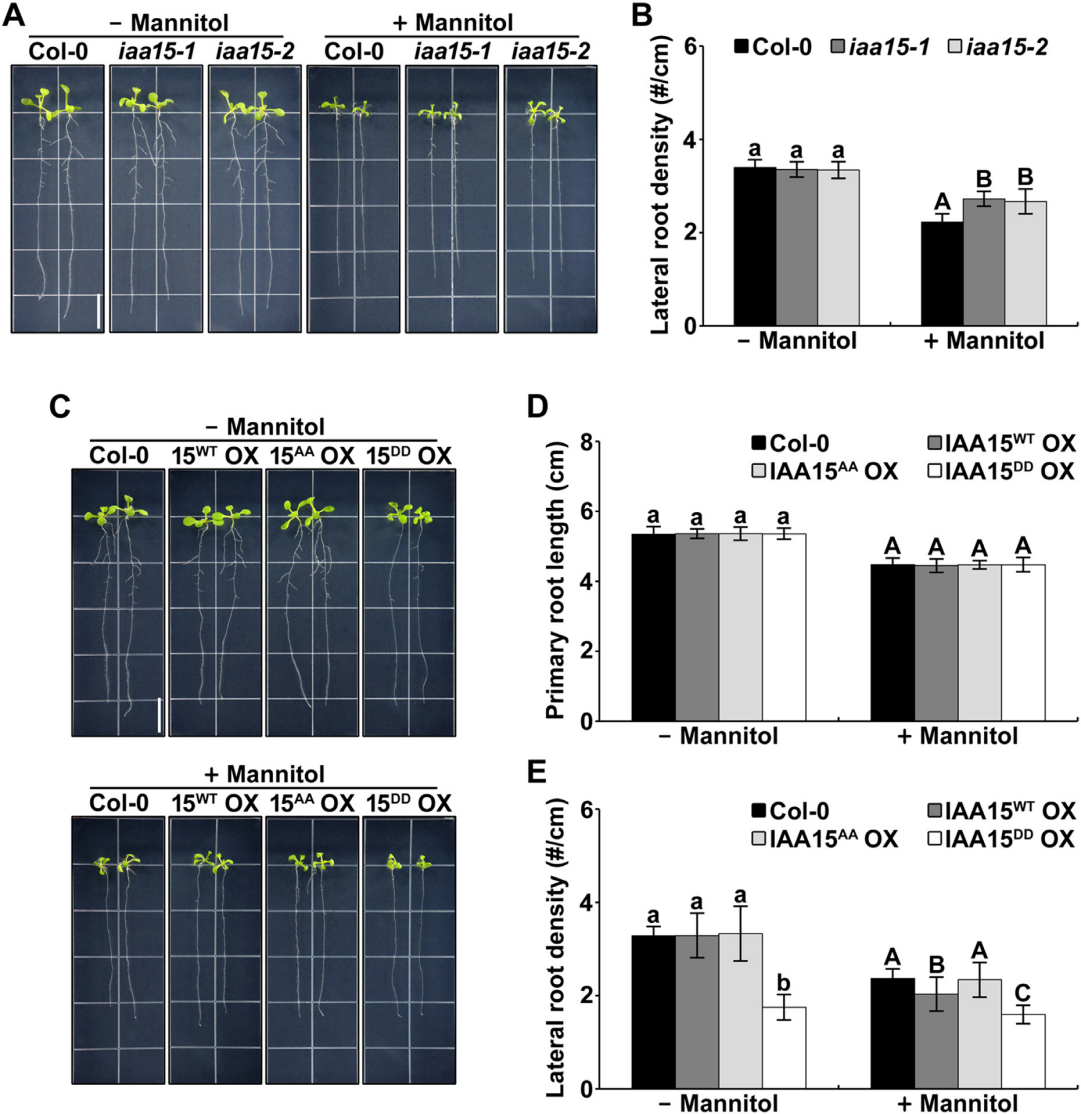
IAA15 is involved in the suppression of lateral root development in response to drought. (**A**) Root growth phenotypes of Col-0, *iaa15-1*, and *iaa15-2* plants. Four-day-old plants were vertically grown on MS plates containing with or without 75 mM mannitol. The plants were photographed after 7 d. The scale bar represents 1 cm. (**B**) Lateral roots density of plants shown in A. The bars indicate the mean ± S.D. (*n* = 20). Different letters represent statistically significant differences between genotypes (*P* < 0.001). (**C**) Root growth phenotypes of Col-0, IAA15^WT^ OX #7, IAA15^AA^ OX #5 and IAA15^DD^ OX #1 plants. Four-day-old plants were vertically grown on MS plates containing with or without 75 mM mannitol. The plants were photographed after 7 d. The scale bar represents 1 cm. (**D, E**) Primary root length (D) and lateral roots density (E) of plants shown in Supplemenatary Figure S7. The bars indicate the mean ± S.D. (*n* = 20). Different letters represent statistically significant differences between genotypes (*P* < 0.01).

To further confirm the physiological function of IAA15 under drought-like conditions, we observed the lateral root phenotypes using two loss-of-function mutants, *iaa15-1* and *iaa15-2* (Supplemental Figure S5A–C). Although these *iaa15* mutants did not produce any visible lateral root phenotype compared to the Col-0 plants under normal conditions (Figure 4A and B; Supplementary Figure S5D), in the presence of mannitol, they obviously showed an increase in the lateral root destiny compared to that of the Col-0 plants (Figure 4A and B; Supplementary Figure S5D), suggesting that IAA15 is a *bona fide* factor involved in the inhibition of lateral root development in response to drought.

To examine the effects of phosphorylation on the physiological function of IAA15, we further constructed transgenic plants overexpressing the nonphosphorylatable mutant *3XFLAG*-*IAA15^AA^* (IAA15^AA^ OX) and the phospho-mimicking mutant *3XFLAG*-*IAA15^DD^* (IAA15^DD^ OX). To select three independent lines of each transgenic plant, we examined the IAA15 transcript and protein expression levels using qPCR and immunoblotting, respectively. All transgenic plants displayed similar overexpression levels of the *IAA15* transcripts (Supplementary Figure S6A). Consistent with Supplementary Figure S3, IAA15 proteins were not detected in the IAA15^WT^ OX and IAA15^AA^ OX plants under normal conditions but were highly detected after the treatment with the proteasome inhibitor MG132 (Supplementary Figure S6B). In contrast, higher levels of IAA15 proteins were detected in the IAA15^DD^ OX plants in the absence and presence of MG132 (Supplementary Figure S6B), indicating that native IAA15^WT^ is unstable but that IAA15^DD^ is stable under normal growth conditions.

Subsequently, we examined the root phenotypes of the IAA15^WT^ OX, IAA15^AA^ OX and IAA15^DD^ OX plants. As shown in Supplementary Figure S6C, the primary root lengths were similar in all transgenic plants under normal growth conditions. In contrast, a visual inspection pointed to shorter and reduced density of the lateral roots in the IAA15^DD^ OX plants, suggesting that the stabilization of IAA15 proteins in the IAA15^DD^ OX plants inhibits lateral root development. Because three independent lines of each transgenic plant showed similar expression patterns and lateral root development (Supplementary Figure S6), we used IAA15^WT^ OX #7, IAA15^AA^ OX #5, and IAA15^DD^ OX #1 as representative lines of each transgenic plant in the following experiments and hereafter refer to them as IAA15^WT^ OX, IAA15^AA^ OX and IAA15^DD^ OX, respectively.

To further investigate whether mannitol inhibits lateral root development through the phosphorylation-mediated stabilization of IAA15, we measured the lateral root density in IAA15 transgenic plants after treatment with mannitol. As expected, the lateral root density in the IAA15^DD^ OX plants was reduced compared to that in the other plants in the absence of mannitol (Figure 4C–E; Supplementary Figure S7). The further reduction of lateral root development in IAA15^DD^ OX plants was not observed in the presence of mannitol (Figure 4E). In addition, we noticed that IAA15^WT^ OX plants were more responsive to mannitol than IAA15^AA^ OX plants (Figure 4C and E; Supplementary Figure S7), indicating that the phosphorylation of IAA15 is required for the suppression of the lateral root development. These results suggest that IAA15 negatively affects lateral root development in response to drought stress.

### Drought-induced accumulation of IAA15 is mediated through MPK phosphorylation

**Figure 5.**
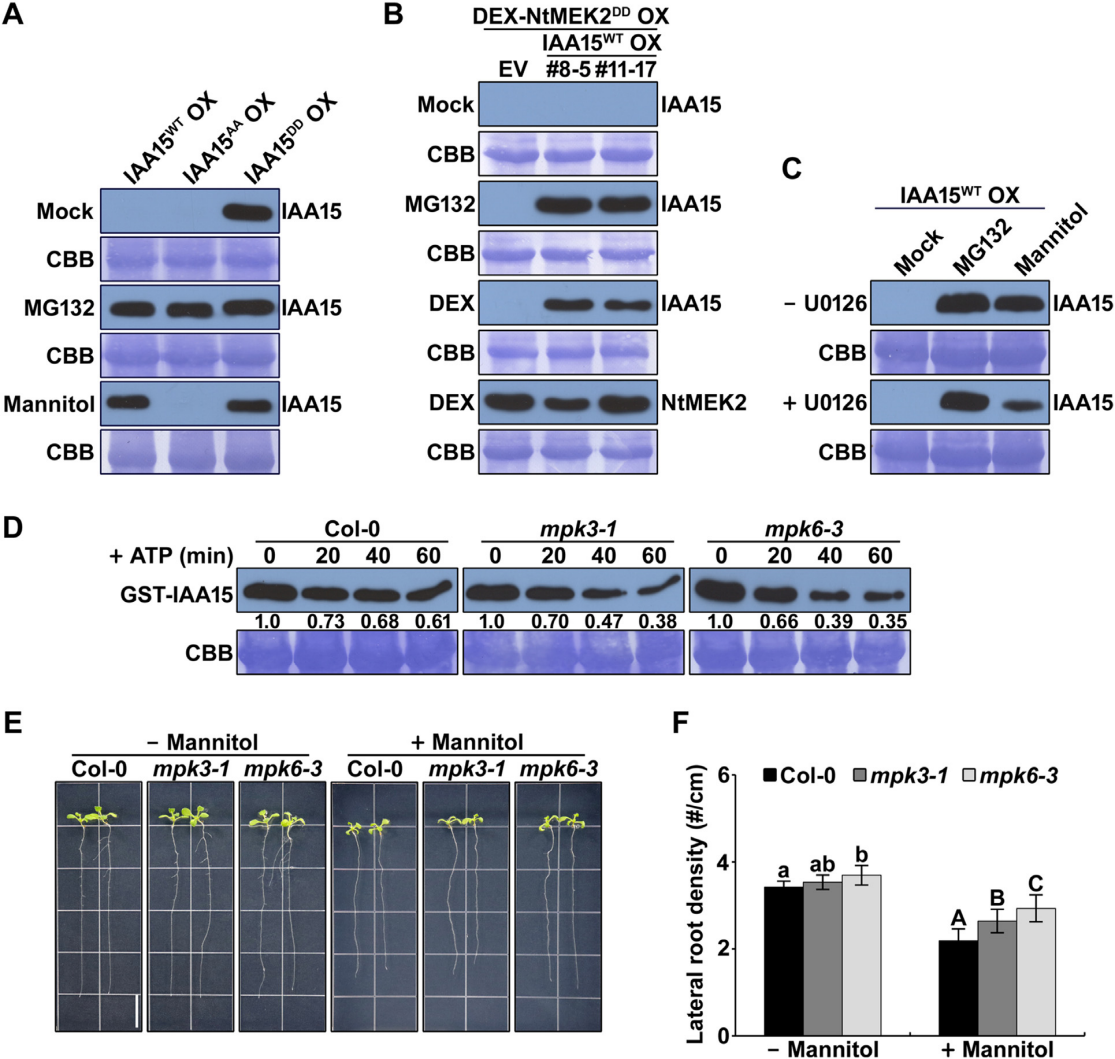
IAA15 was stabilized by mannitol through the phosphorylation of MPK cascades. (**A**) Protein levels of IAA15 in transgenic plants in mock, MG132, and mannitol treatments. IAA15 proteins were detected by immunoblotting with anti-Flag antibodies. The Rubisco band stained by CBB is shown to verify similar amounts of loaded protein (lower panel). (**B**) Stabilization of IAA15 by the expression of constitutively active tobacco MEK2 (NtMEK2^DD^). Immunoblotting analyses were performed in double transgenic plants overexpressing HA-fused IAA15^WT^ and Flag-fused NtMEK2^DD^. The plants were treated with mock, MG132, and DEX treatments. IAA15 and NtMEK2^DD^ proteins were detected by immunoblotting with anti-HA and anti-Flag antibodies, respectively. The Rubisco band stained by CBB is shown to verify similar amounts of loaded protein (lower panel). (**C**) Mannitol-induced stabilization of IAA15 was inhibited by the MEK inhibitor U0126. IAA15^WT^ OX plants pretreated with or without U0126 were subjected to MG132 and mannitol treatments. IAA15 was detected by immunoblotting with anti-Flag antibodies. The Rubisco band stained by CBB is shown to verify similar amounts of loaded protein (lower panel). (**D**) MPK3 and MPK6 inhibit IAA15 degradation in a cell-free degradation assay. Equal amounts of total proteins extracted from 2-week-old Col, *mpk3-1*, and *mpk6-3* plants were incubated with recombinant GST-IAA15 protein in the presence of ATP. IAA15 was detected with anti-GST antibody. The Rubisco band stained by CBB is shown to verify similar amounts of used total protein (lower panel). (**E**) Root growth phenotypes of Col-0, *mpk3-1*, and *mpk6-3* plants. Four-day-old plants were vertically grown on MS plates containing with or without 75 mM mannitol. The plants were photographed after 7 d. The scale bar represents 1 cm. (**F**) Lateral root density of plants shown in E. The bars indicate the mean ± S.D. (*n* = 20). Different letters represent statistically significant differences between genotypes (*P* < 0.05).

Most Aux/IAA proteins are polyubiquitinated by the SCF^TIR1/AFB^-E3 ubiquitin ligase complex and rapidly degraded by 26S proteasome-mediated proteolysis under normal conditions. We showed that IAA15^DD^ proteins became stable even under normal conditions (Figure 5A; Supplementary S6B). Furthermore, the IAA15^WT^ protein was phosphorylated and stabilized by the treatment with mannitol, but IAA15^AA^ protein was not (Figures 3D and 5A). Therefore, we hypothesized that phosphorylation by MPKs might stabilize the IAA15 protein. To evaluate this hypothesis, we constructed double transgenic plants overexpressing hemagglutinin (HA)-tagged IAA15^WT^ driven by the *35S* promoter and NtMEK2^DD^, a constitutively active tobacco MEK2 known to activate MPK3 and MPK6 of Arabidopsis, driven by a dexamethasone (DEX)-inducible promoter (HA-IAA15^WT^/Flag-NtMEK2^DD^ OX) (38,54). Two independent transgenic lines were treated with MG132 to inhibit the degradation of IAA15^WT^ protein or DEX to activate the MPK cascade by the induction of NtMEK2^DD^. As expected, the IAA15^WT^ protein was not detected in the absence of MG132 (mock) but was highly detected after the treatment with MG132 or DEX (Figure 5B; Supplementary Figure S8). Furthermore, high activation of MPK3 and MPK6 was detected by DEX-induced NtMEK2^DD^ in the transgenic plants (Supplementary Figure S8), which corroborates our finding that MPK phosphorylation increases the stability of the IAA15 protein.

Since the activity of MPKs and the stability of IAA15 were increased by the treatment with mannitol (Figures 3C, D and 5A), we hypothesized that the stabilization of IAA15 in response to mannitol is mediated by the activities of MPK. To confirm this possibility, we applied an MKK inhibitor, U0126, during the mannitol-mediated stabilization of IAA15. As a result, the mannitol-mediated stabilization of IAA15 was significantly reduced by the cotreatment with U0126 (Figure 5C), indicating that active MPKs are essential for stabilizing IAA15 in response to drought. To further confirm that both MPK3 and MPK6 are involved in the stabilization of IAA15 in response to mannitol, we performed cell-free protein degradation assays using a recombinant GST-IAA15 protein and total cell extracts from Col-0, *mpk3*, and *mpk6* plants treated with mannitol. As previously described, we added ATP to enhance the protein degradation rate in this assay (46,47). Although the GST-IAA15 fusion proteins were gradually degraded by the incubation with the total cell extracts from all plants (Figure 5D), reduced levels of fusion proteins were detected in the *mpk3* and *mpk6* single mutants than in Col-0 (Figure 5D), indicating that the mannitol-mediated stabilization of IAA15 is achieved by the redundant functions of MPK3 and MPK6.

Since IAA15 was stabilized by the phosphorylation of MPK3 and MPK6, we postulated that MPK3 and MPK6 are also negatively involved in lateral root development in response to mannitol. Because MPK6 was reported to act as a negative regulator of lateral root development (35,55), we analyzed lateral root development in *mpk3* and *mpk6* plants in the absence and presence of mannitol. As a result, the *mpk6* plants showed increased lateral root density compared with the Col-0 plants under normal conditions (Figure 5E and F; Supplementary Figure S9A). Moreover, the *mpk3* plants exhibited a slight increase in lateral roots. In the presence of mannitol, both the *mpk3* and *mpk6* plants showed increased lateral roots than the Col-0 plants (Figure 5E and F; Supplementary Figure S9), implying that both MPK3 and MPK6 are involved in the suppression of lateral roots in response to drought. These results demonstrate that the stabilization of IAA15 mediated by MPK phosphorylation plays a key role in the inhibition of lateral root development in response to drought.

### Phosphorylation of IAA15 attenuates its polyubiquitination

The rapid auxin-induced degradation of Aux/IAA proteins is triggered by their polyubiquitination via the SCF^TIR1/AFB^-E3 ubiquitin ligase complex pathway. To understand how the phosphorylation by MPKs increases IAA15 stability, we postulated two possible mechanisms. First, the phosphorylation of IAA15 may affect its interaction with TIR1. To test the first possibility, we analyzed the interaction between IAA15^WT^ or its mutants and TIR1 using yeast two-hybrid assays. In the yeast growth and *β*-galactosidase assays, we did not observe differences in the interactions between IAA15^WT^ or its mutants and TIR1 in the absence and presence of auxin (Supplementary Figure S10), demonstrating that phosphorylation does not affect the interaction between IAA15 and TIR1.

**Figure 6.**
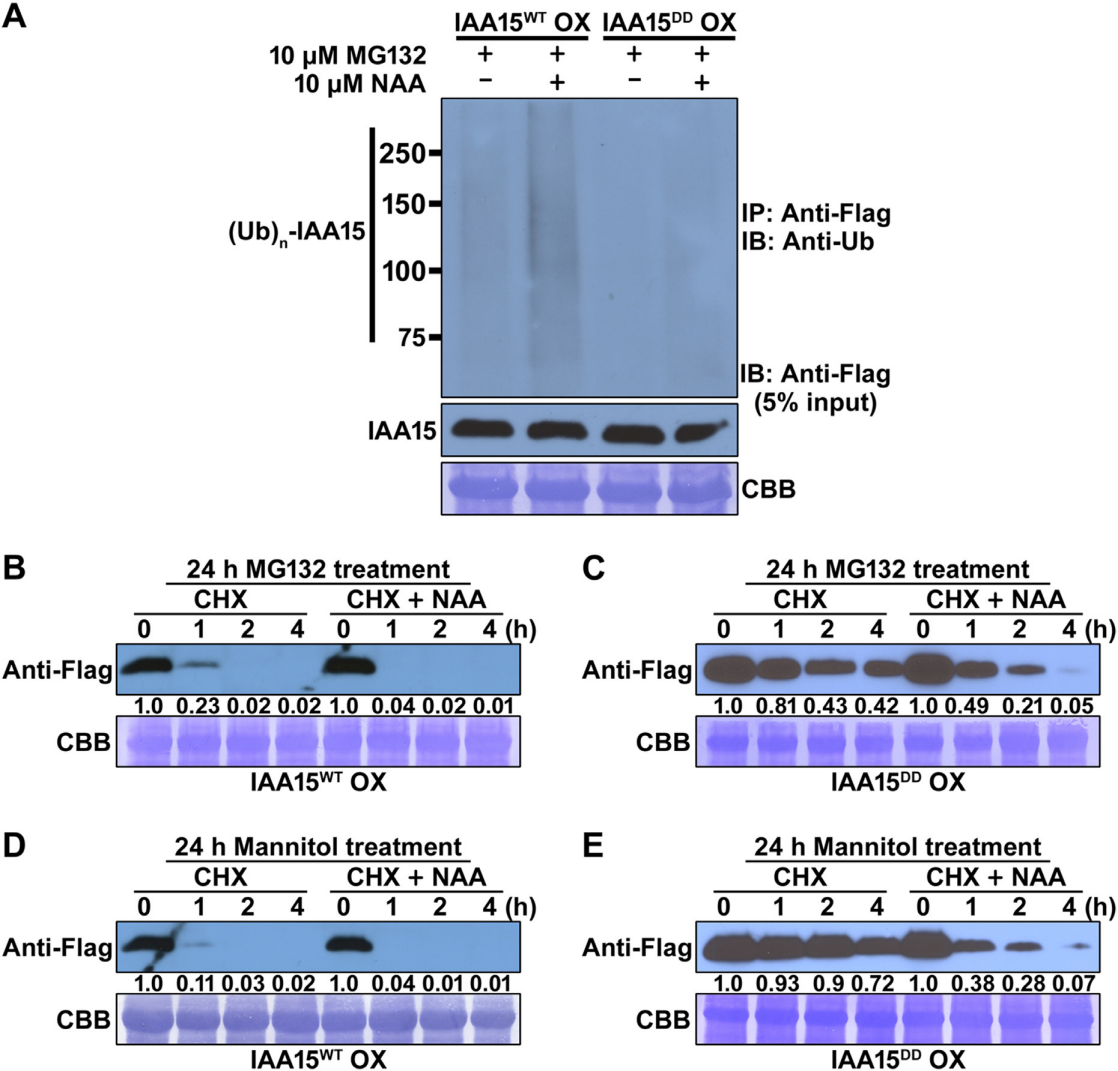
The IAA15^DD^ mutant is more stable than IAA15^WT^. (**A**) Auxin-induced polyubiquitination of IAA15^WT^ and IAA15^DD^ proteins. Polyubiquitinated IAA15 proteins were detected by immunoblotting (IB) with an anti-ubiquitin (anti-Ub) antibodies after immunoprecipitation (IP) with anti-Flag antibody from IAA15^WT^ OX and IAA15^DD^ OX plants treated with or without NAA in the presence of MG132 (upper panel). IAA15 proteins were detected by immunoblotting (IB) with anti-Flag antibody (lower panel, 10% input). (**B–E**) Protein stabilities of IAA15^WT^ and IAA15^DD^*in planta*. Two-week-old plants of IAA15^WT^ OX and IAA15^DD^ OX pretreated with 10 μM MG132 (B, C) and 200 mM mannitol (D, E) for 20 h were applied with 1 mM cycloheximide (CHX) and 10 μM NAA for the indicated times. Total proteins extracted from the transgenic plants were subjected to immunoblotting with anti-Flag antibodies. The Rubisco band stained by CBB is shown to verify similar amounts of loaded protein (lower panel).

Second, phosphorylation may affect the polyubiquitination of IAA15 by the SCF^TIR1/AFBs^-E3 ubiquitin ligase complex. To examine the second possibility, we evaluated the polyubiquitination efficiency in IAA15^WT^ and IAA15^DD^ OX plants. To prevent the degradation of polyubiquitinated IAA15, we pretreated IAA15^WT^ and IAA15^DD^ OX plants with MG132 for 20 h and then treated them with or without NAA for 4 h. The accumulated IAA15 proteins were immunoprecipitated with anti-Flag antibodies from the total protein extracts. Immunoblotting was performed with anti-ubiquitin (anti-Ub) antibodies to measure polyubiquitinated IAA15. In response to MG132, the inputs of the IAA15 proteins were similar between the IAA15^WT^ and IAA15^DD^ OX plants (middle panel, Figure 6A). In the IAA15^WT^ OX plants, higher molecular weight bands, possibly polyubiquitinated IAA15, were visible in the absence of NAA, and the bands were significantly increased in the presence of NAA (upper panel, Figure 6A). In contrast, the levels of polyubiquitinated IAA15 in the IAA15^DD^ OX plants were lower (in both the absence and presence of NAA) than those in the IAA15^WT^ OX plants (upper panel, Figure 6A), indicating that phosphorylation suppresses the polyubiquitination of IAA15.

Subsequently, we performed a protein turnover assay in IAA15^WT^ and IAA15^DD^ OX plants to evaluate the stability of phosphorylated and unphosphorylated IAA15. To inhibit the degradation of IAA15 proteins by the proteasome, these plants were pretreated with MG132 for 24 h and then treated with cycloheximide (CHX) or CHX plus NAA for 4 h. CHX was used to prevent the *de novo* synthesis of proteins in the plants. As a result, the IAA15^WT^ proteins rapidly disappeared within 2 h regardless of the NAA treatment and were almost undetectable after 4 h (Figure 6B). In contrast, IAA15^DD^ was highly detected regardless of the NAA treatment (Figure 6C). Furthermore, we obtained similar results after the treatment with mannitol (Figure 6D and E). These results strongly suggest that IAA15 phosphorylated by MPK is stabilized by the inhibition of SCF^TIR1/AFB^-E3 ubiquitin ligase complex-mediated polyubiquitination.

### Stabilized IAA15 inhibits lateral root development through the transcriptional downregulation of *LBD* genes

**Figure 7.**
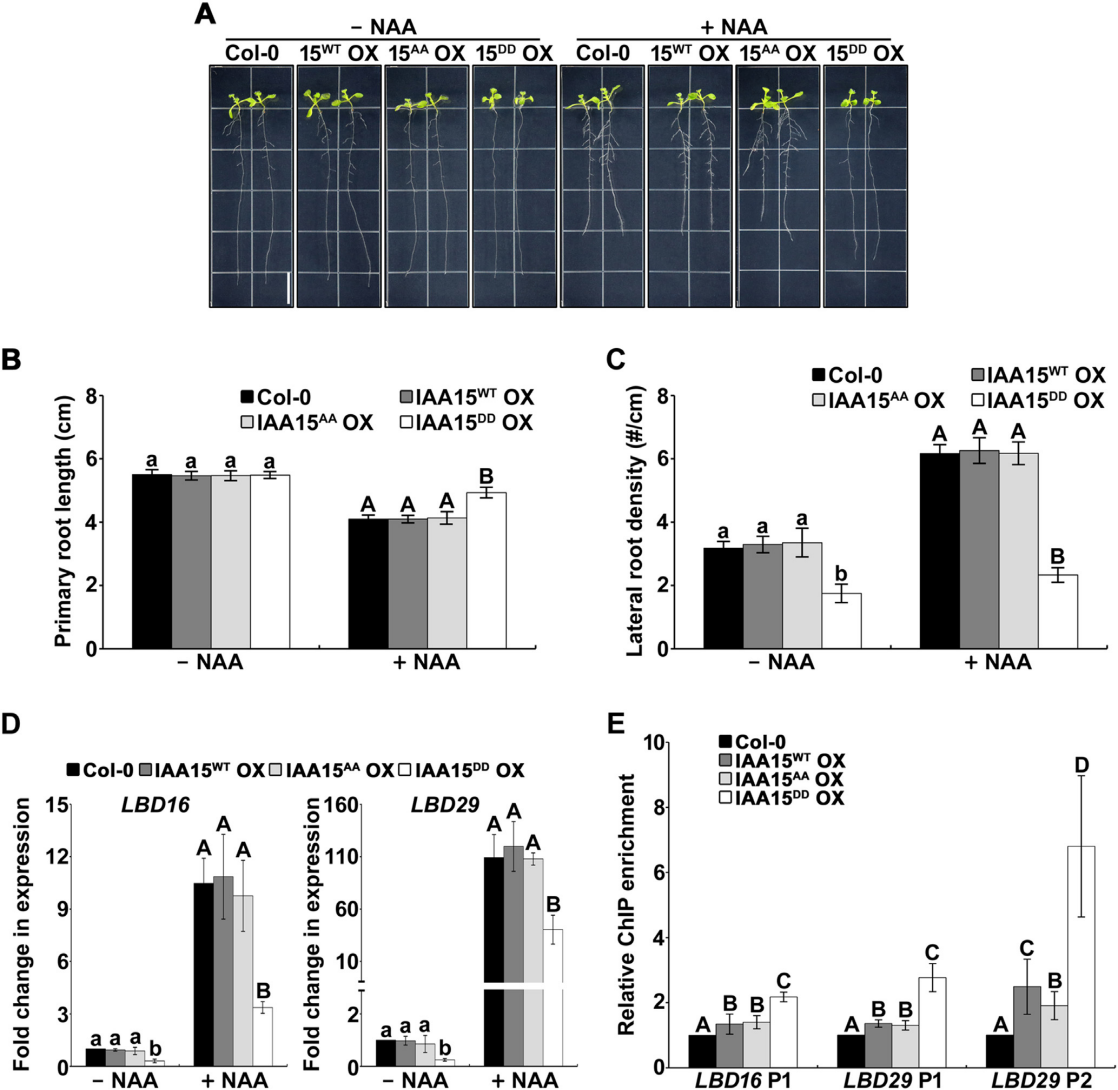
Auxin-insensitive phenotypes and decreased expression of *LBD* genes in IAA15^DD^ OX plants. (**A**) Effects of auxin on the root growth of Col-0, IAA15^WT^ OX, IAA15^AA^ OX, and IAA15^DD^ OX plants. Shown are 2-week-old plants vertically grown on MS plates containing 25 nM NAA. The scale bar represents 1 cm. (**B, C**) Primary root length (B) and lateral root density (C) of plants shown in Supplementary Figure S12. The bars indicate the mean ± S.D. (*n* = 20). Different letters represent statistically significant differences between genotypes (*P* < 0.001). (**D**) Expression of *LBD* genes was down-regulated in IAA15^DD^ OX plants. Transcript levels of *LBD16* and *LBD29* were measured by qPCR using specific primers. The bars indicate the mean ± S.D. (*n* = 3). Different letters represent statistically significant differences between genotypes (*P* < 0.01). (**E**) *In vivo* binding of IAA15 to the promoters of *LDB16* and *LBD29*. ChIP assays were performed using anti-Flag antibodies with chromatin prepared from transgenic plants. ChIP-DNA was applied to qPCR using primers specifically targeting *LBD16* promoter (P1) and *LBD29* promoter (P1 and P2) regions that contain auxin-responsive elements. The bars indicate the mean ± S.D. (*n* = 3). Different letters represent statistically significant differences between genotypes (*P* < 0.05).

Since Aux/IAAs are repressors of auxin signaling, we assumed that stabilized IAA15 represses auxin signaling in the roots. To evaluate this assumption, we measured GUS activity in double transgenic plants overexpressing IAA15^WT^, IAA15^AA^ or IAA15^DD^ on a *DR5*::*GUS* background. GUS activity was significantly reduced in the emerged lateral roots of the IAA15^DD^ OX plants compared to those of the IAA15^WT^ and IAA15^AA^ OX plants (Supplementary Figure S11). In addition, the IAA15^DD^ OX plants showed a longer primary root length and reduced lateral root density in response to NAA compared to the other plants (Figure 7A-C; Supplementary Figure S12), indicating that the IAA15^DD^ OX plant is less sensitive to NAA. These results demonstrate that the stabilization of the IAA15 protein by phosphorylation represses the auxin response in the roots.

Since reduced lateral root development in a gain-of-function mutant of IAA15 is caused by the suppressed transcription of *LBD* genes (25), we hypothesized that the reduced lateral root phenotype of the IAA15^DD^ OX plants might be caused by the downregulation of *LBD* genes. Therefore, we examined the transcript levels of *LBD16* and *LBD29* in IAA15^DD^ OX plants. Consistent with our observations, we observed that the transcript levels of *LBD16* and *LBD29* were decreased in the IAA15^DD^ OX plants regardless of the NAA treatment (Figure 7D), underscoring that the reduced lateral roots in the IAA15^DD^ OX plants are caused by the transcriptional downregulation of *LBD* genes.

To examine the binding of IAA15 to the promoters of the *LBD16* and *LBD29* genes, we performed a chromatin immunoprecipitation (ChIP) assay. There are one *(LBD16* P1) and two (*LBD29* P1 and *LBD29* P2) auxin-responsive elements (AuxREs) in the promoters of the *LBD16* and *LBD29* genes, respectively. The enrichment of *LBD* promoters was significantly increased in the IAA15^DD^ OX plants (Figure 7E). Unexpectedly, this enrichment was also slightly increased in the IAA15^WT^ and IAA15^AA^ OX plants, probably due to overexpression. Since IAA15 is believed to inhibit the expression of *LBD16* and *LBD29* through the formation of a complex with ARF7 and ARF19 (25), we investigated whether phosphorylation affects the interaction between IAA15^WT^ or its mutant and ARFs. As expected, phosphorylation did not change the interactions between IAA15 and ARFs in the yeast two-hybrid assays because Aux/IAAs interact with ARFs via domains III and IV of their C-terminal (Supplementary Figure S13). These results demonstrate that the stabilization of IAA15 by phosphorylation downregulates the transcription of *LBD* genes through interactions with ARF7 and ARF19.

## DISCUSSION

Plant developmental plasticity is critical for plant survival under various environmental stresses. To induce tolerance in response to drought, plants change their root system architectures (RSAs), including reduced lateral roots. Therefore, understanding the molecular mechanism by which drought affects lateral root development could help develop drought-tolerant plants. This study demonstrates that MPK-mediated phosphorylation in response to drought induces the stabilization of IAA15, which inhibits lateral root development.

### IAA15 is phosphorylated by MPK3 and MPK6

MPK cascades are involved in various physiological responses as conserved processes in all eukaryotes. However, only a limited number of MPK substrates have been identified in Arabidopsis. This study showed that IAA15 is phosphorylated by drought-responsive MPK3 and MPK6 through direct interaction (Figures 2 and 3). The *in vitro* and *in vivo* phosphorylation of IAA15 was verified using different kinase assays and phos-tag assays (Figure 3). Based on our results, we suspect that the phosphorylation of Aux/IAAs could be a major posttranslational modification (PTM) that regulates their activities. Previously, several Aux/IAAs were predicted to be substrates of MPKs (37). In addition, other Aux/IAAs were reported to be phosphorylated by different kinases, such as phytochrome A (phyA), transmembrane kinase 1 (TMK1), and MPK14. SHY2/IAA3, AXR3/IAA17, IAA1, IAA9 and *Ps*-IAA4 were phosphorylated by recombinant oat PhyA (56). Noncanonical IAA32 and IAA34 are phosphorylated by the C-terminus of TMK1, which promotes apical-hook development (57). Similar to our findings, MPK14 has been shown to phosphorylate noncanonical IAA33 (58). In addition, PhyB and CRY1 are expected to phosphorylate the Aux/IAA proteins because PhyB and CRY1 were found to interact with IAA7, IAA12 and IAA17 (59).

Furthermore, in this study, we showed that several other Aux/IAA proteins are substrates of MPKs using an *in vitro* phosphorylation assay (Supplementary Figure S2). Further studies investigating the phosphorylation of Aux/IAAs by MPKs could be helpful in elucidating the involvement of MPK-Aux/IAA modules in plant growth and development under environmental stresses.

### Phosphorylation of IAA15 improves protein stability by inhibiting polyubiquitination

Since most Aux/IAA proteins are polyubiquitinated by the SCF^TIR1/AFB^-E3 ubiquitin ligase complex and degraded by 26S proteasome-mediated proteolysis (15,16), the physiological functions of Aux/IAAs are believed to be mainly regulated by their protein stability in response to environmental cues. Three different mechanisms help Aux/IAAs achieve stability under environmental stresses. First, decreasing the auxin concentration can contribute to the stabilization of Aux/IAAs. High temperatures inhibit the expression of the auxin biosynthesis-related genes *YUC2* and *YUC6*, leading to a decrease in the local auxin levels (60,61). Therefore, the decreased auxin concentration due to environmental stress results in the stabilization of Aux/IAAs through the inhibition of auxin-mediated polyubiquitination. Second, Aux/IAAs can be stabilized by the transcriptional repression of the auxin receptors *TIR1/AFBs*, which are essentially required for the polyubiquitination of Aux/IAAs. It has been reported that flagellin-derived peptides and drought facilitate the stabilization of Aux/IAA through the accumulation of *miRNA393*, which suppresses the transcription of *TIR1/AFB*s (62,63). Third, Aux/IAAs can be stabilized by protein−protein interactions with other proteins. Recently, several studies have reported that some Aux/IAAs are stabilized by both red-light and blue-light receptors through direct interactions (59,64). In addition to these three mechanisms, we uncovered that the phosphorylation of drought-responsive MPKs induces the stability of IAA15 (Figures 3 and 5). Phosphorylation affects the activity, stability, localization, and interaction of proteins (65). Similar to IAA15, the stability of noncanonical IAA33 is increased by the phosphorylation of MPK14 (58). Moreover, the stabilities of ERF6 and LIP5, which are involved in pathogen and salt resistance, respectively, are increased by the phosphorylation of MPK3 and MPK6 (66–68).

It has been reported that protein stability is regulated by combinations of PTMs (69). For example, the stability of ICE1, a positive regulator of cold signaling, is tightly regulated by combinations of ubiquitination, SUMOylation, and phosphorylation (70–73). NPR1 and ABI5, which are key regulators of plant immunity and ABA signaling, respectively, also represent other examples in which stability is regulated by multiple PTMs (74–76). Similarly, we revealed that the phosphorylation of drought-responsive MPKs inhibits the polyubiquitination of IAA15 (Figure 6A). The analysis of the amino acid sequence of the IAA15 protein revealed that two phosphorylation sites are distantly located (Ser-2 and Thr-28) from the SCF^TIR1/AFB^ docking motif (Domain II) but relatively close to the putative ubiquitination site (Supplementary Figure S14), indicating that covalent PTM by phosphate groups at two phosphorylation sites of IAA15 might interfere with the attachment of the ubiquitin moiety to the putative ubiquitination sites by charge or steric hindrance.

This explanation can be extended and applied to other IAAs. We performed a multiple alignment analysis to estimate whether the phosphorylation sites are conserved in other Aux/IAA families. The N-terminal amino acids (including domains I and II) of 9 canonical Aux/IAAs were aligned (Supplementary Figure S14). Most potential MPK phosphorylation sites, Ser and Thr residues in S/T-P motifs, were observed in the N-terminus of Aux/IAAs: IAA4 (Ser-57), IAA7 (Ser-26), IAA8 (Ser-16, Ser-53, Ser-74, Thr-77, and Ser-135), IAA12 (Ser-63), IAA14 (Ser-21), IAA15 (Ser-2 and Thr-28), IAA18 (Ser-11 and Ser-80), IAA19 (Ser-64), IAA28 (Ser-168), IAA29 (Ser-10) and IAA34 (Ser-44). Since IAA34 is a noncanonical isoform, and IAA28 has a predicted phosphorylation site distant from the N-terminus, we excluded them from the alignment analysis. As shown in Supplementary Figure S14, although the positions of the phosphorylation sites were not conserved among phosphorylated Aux/IAAs, most sites were located near their potential ubiquitination sites, suggesting that the common mechanism may regulate the stabilities of these IAAs. In the future, to understand the molecular mechanism by which phosphorylation prevents the polyubiquitination of IAA15, a structural analysis of phosphorylated and unphosphorylated IAA15 is needed.

### MPK-IAA15 module suppresses lateral root development

In response to external stimuli, MPK cascades transduce signals for appropriate cellular responses. Two controversial studies revealed the physiological roles of MPKs in lateral root development. MPKs are known to be positively involved in lateral root development via noncanonical auxin signaling pathways under normal conditions (77–79). Consistently, MPK14 has been reported to promote lateral root development by regulating ERF13 (80). In contrast, it has also been reported that MPK6 is negatively involved in lateral root development in Arabidopsis (35,55). In this study, we also showed that MPK3 and MPK6 are negatively involved in lateral root development by using two single MPK mutants under drought conditions (Figure 5E and F). Moreover, we found that the phosphorylation of IAA15 plays a negative role in lateral root development under drought conditions at downstream of MPKs (Figures 3–5). These results demonstrate that the MPK-IAA15 module negatively regulates lateral root development through the inhibition of auxin signaling under drought conditions.

Most gain-of-function mutants of *Aux*/*IAA*, including *IAA3*/*SHY2*, *IAA14*/*SLR1-1*, *IAA19*/*MSG2* and *IAA28*, show pleiotropic auxin deficiency-related phenotypes (21–24). Similarly, the gain-of-function mutant of IAA15, IAA15^P78S^ OX, also showed pleiotropic phenotypes, including a decreased number of lateral roots (25). However, the IAA15^DD^ OX plants showed a reduced lateral root number (Figure 4C and E). These phenomena raise the question of why the IAA15^DD^ OX plants did not show the pleiotropic phenotypes. To understand the difference between the IAA15^DD^ OX and IAA15^P78S^ OX plant phenotypes, we characterized the molecular differences between IAA15^DD^ or IAA15^P78S^ proteins and IAA15^WT^ protein. First, we examined the *in planta* polyubiquitination of these proteins. IAA15^DD^ was less polyubiquitinated than IAA15^WT^ but more polyubiquitinated than IAA15^P78S^ (Supplementary Table S4). Second, the stabilities of these proteins were determined. Expectedly, in proportion to their polyubiquitination level, IAA15^WT^ was very unstable, IAA15^DD^ was stable, and IAA15^P78S^ was highly stable (Supplementary Table S4). Third, the lateral root development of these transgenic plants was investigated. As a result, we found that IAA15^DD^ OX showed decreased later roots and IAA15^P78S^ OX showed much more decreased lateral roots compared to IAA15^WT^ OX in proportion to their protein stabilities (Supplementary Table S4). Based on these results, we may conclude that IAA15^P78S^ is more stable than IAA15^DD^. Subsequently, we need to explain why IAA15^P78S^ is more stable than IAA15^DD^. First, we can suspect that different molecular mechanisms underly their protein stabilization. It seems that the gain-of-function mutation almost completely inhibits the interactions between Aux/IAA and TIR1 because the mutation is located in the degron motif, resulting in a high accumulation of corresponding Aux/IAA protein. This high accumulation of Aux/IAA in gain-of-function mutants may cause pleiotropic auxin deficiency-related phenotypes. In contrast, the phospho-mimicking mutation only inhibits the polyubiquitination of IAA15 by the SCF^TIR1/AFBs^-E3 ubiquitin ligase complex (Figure 6A; Supplementary Figure S10) because the mutation is located moderately away from the degron motif. Compared to IAA15^WT^, IAA15^DD^ was more stable but degraded by the increase in auxin (Figure 6B-E), suggesting that the phospho-mimicking mutant is a moderately stabilized form. Therefore, we may explain why IAA15^DD^ OX plants only exhibit a specific phenotype in lateral roots instead of pleiotropic phenotypes.

Subsequently, we found that stabilized IAA15 represses lateral root development in response to drought (Figure 4), while drought stress inhibits both primary and lateral root development (Figure 1). These results suggest that other signaling components, including other IAAs, are involved in the suppression of primary root development in response to drought. Therefore, the identification of other signaling components that play roles in the suppression of primary root development could be performed to elucidate how drought stress suppresses both primary and lateral root development.

### Can the MPK-IAA15 module contribute to drought tolerance by inhibiting lateral root development?

Growth-defense tradeoffs occur in plants due to limited resources. This tradeoff requires the prioritization of either growth or defense depending on environmental conditions (81). In general, stress tolerance is associated with the inhibition of plant growth and development. Drought is known to inhibit auxin responses by modulating auxin biosynthesis, transport, and signaling (82). Therefore, it was speculated that inhibiting auxin-mediated growth by stabilized Aux/IAA contributes to acquiring tolerance to environmental stresses. Previously, several studies of Aux/IAA genes demonstrated that stress tolerance requires the repression of auxin signaling by Aux/IAA proteins. For example, the recessive triple mutants *iaa5-1*/*iaa6-1*/*iaa19-1* are hypersensitive to desiccation stress (83). In contrast, overexpression of drought-induced *Aux/IAA* improved drought tolerance in rice (84). The upregulation of *IAA5*, *IAA6* and *IAA19* repressor genes by CBF1 and DREB2A/B enhanced drought tolerance (83,85). These phenomena were similarly found in crosstalk between SA and auxin signaling for pathogen resistance (86). The gain-of-function mutant *axr2*/*iaa7* shows increased resistance to pathogens due to the repression of auxin-mediated plant growth. Furthermore, SA-mediated inhibition of auxin signaling partially contributes to pathogen resistance in plants by stabilizing IAA7 proteins (86). Similarly, IAA15^DD^ OX plants exhibited significantly enhanced drought tolerance (Supplementary Figure S15). This result suggests the possibility that IAA15^DD^ OX plants might show enhanced drought tolerance due to a reduced lateral root phenotype. To address this possibility, we aim to further investigate the various physiological responses to drought stress, including the measurement of respiration and the expression of drought-responsive genes.

**Figure 8.**
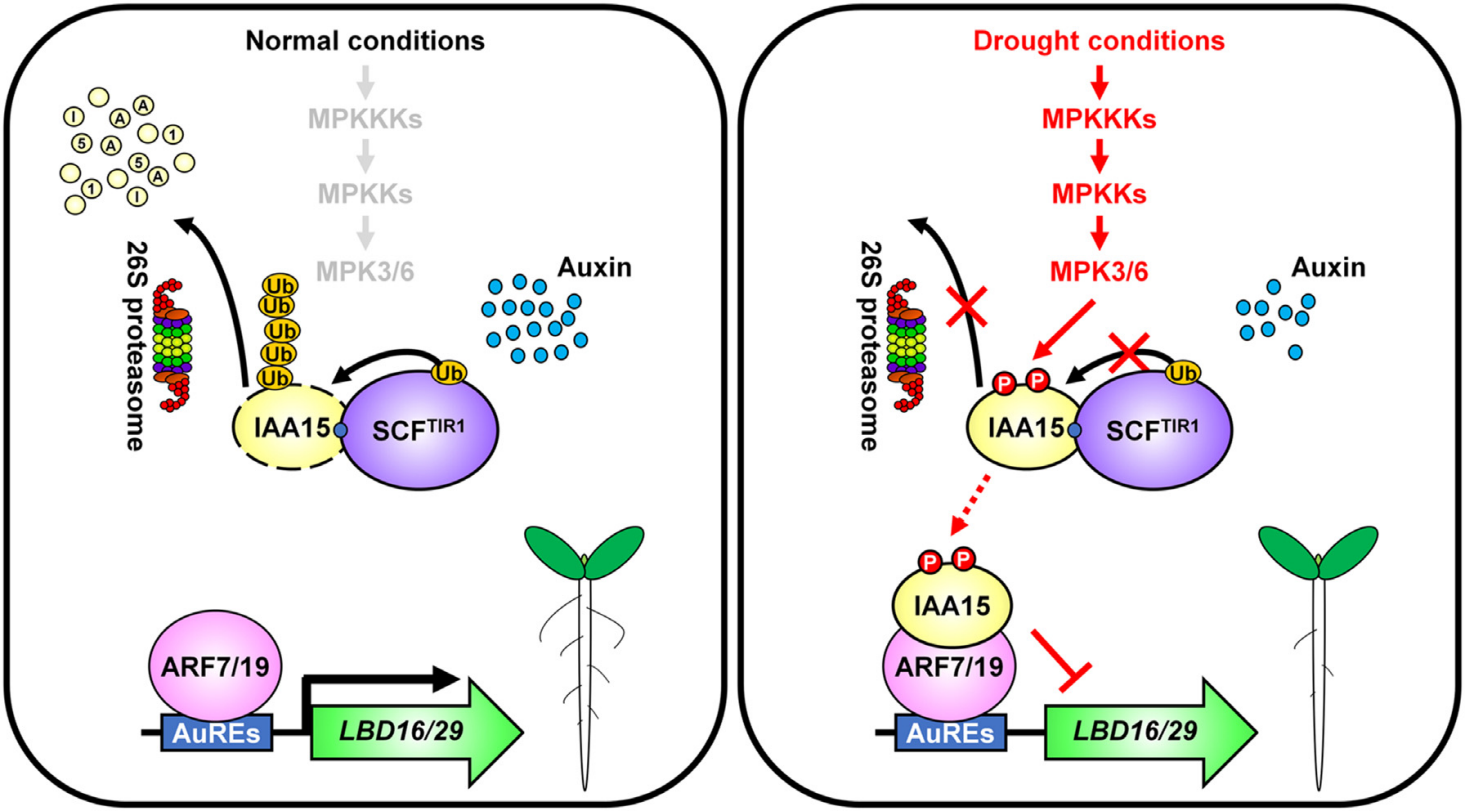
Proposed model for the suppression of lateral root development in response to drought by the phosphorylation of IAA15. Under normal conditions, auxin promotes 26S proteasome-mediated degradation of polyubiquitinated IAA15 by the SCF^TIR1/AFBs^-E3 ubiquitin ligase complex. Free ARF7 and ARF19 activate the transcription of the *LBD16* and *LBD29* genes, which induce normal lateral root development. Under drought conditions, activated MPK3 and MPK6 phosphorylate IAA15. Phosphorylated IAA15 is stabilized through inhibition of polyubiquitination by the SCF^TIR1/AFB^-E3 ubiquitin ligase complex. Stabilized IAA15 suppresses the transcription of *LBD* genes by inhibiting ARF7 and ARF19 transcriptional activities, which suppresses lateral root development.

In conclusion, our observations reveal the prominent role of the MPK-IAA15 module in lateral root development in response to drought (Figure 8). Under normal conditions, IAA15 is polyubiquitinated by the auxin-mediated SCF^TIR1/AFB^-E3 ubiquitin ligase and subsequently undergoes rapid degradation via 26S proteasome assembly, resulting in normal lateral root development. However, under drought conditions, MPKs stabilize IAA15 through phosphorylation, consequently inhibiting the transcriptional expression of *LBD* genes, causing reduced lateral root development. This study is critical for understanding how Aux/IAAs regulate growth and defense tradeoffs in plants. Further studies are needed to elucidate the biological functions of other stabilized Aux/IAAs under various environmental stresses.

## DATA AVAILABILITY

Sequence data from this article can be found in the Arabidopsis Genome Initiative or GenBank/EMBL databases under the following accession numbers: *IAA15*, At1g80390; *MPK3*, At3g45640; *MPK4*, At4g01370; *MPK6*, At2g43790; *LBD16*, At2g42430; *LBD29*, At3g58190; *ARF7*, At5g20730; *ARF19*, At1g19220; *TIR1*, AT3G62980 and *Tubulin*, At5g62690.

## Supplementary Material

gkac798_Supplemental_File

## Notes

Present address: Nhan Thi Nguyen, Institute of Environmental Technology, Vietnam Academy of Science and Technology, 18 HoangQuoc Viet, Cau Giay, Hanoi, 11355, Vietnam.
