# Supplementary material for: Phosphorylation of the auxin signaling transcriptional repressor IAA15 by MPKs is required for the suppression of root development under drought stress in Arabidopsis
Source: Nucleic Acids Res. 2022 Sep 26;50(18):10544–61. doi: 10.1093/nar/gkac798 (PMC9561270; doi:10.1093/nar/gkac798)
Supplement: gkac798_Supplemental_File [file gkac798_supplemental_file.docx]

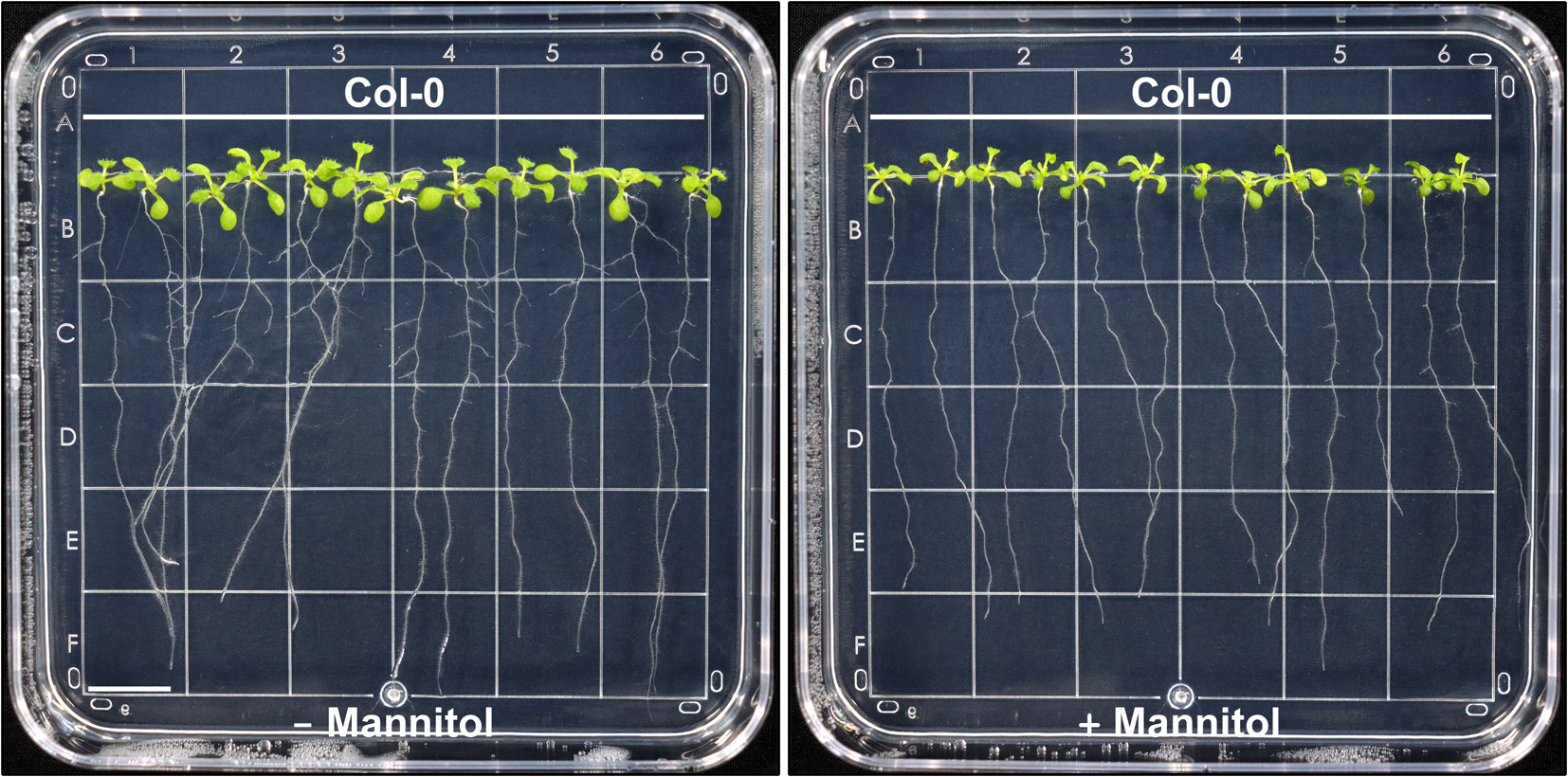


**Supplementary Figure S1.** Phenotype of Col-0 plants in response to drought. Root morphologies of Col-0 plants grown vertically on MS plates containing with or without 75 mM mannitol. Four-day-old plants grown on MS medium were transferred to the plates shown. The plants were photographed at 7 d after the transfer. The scale bar represents 1 cm.


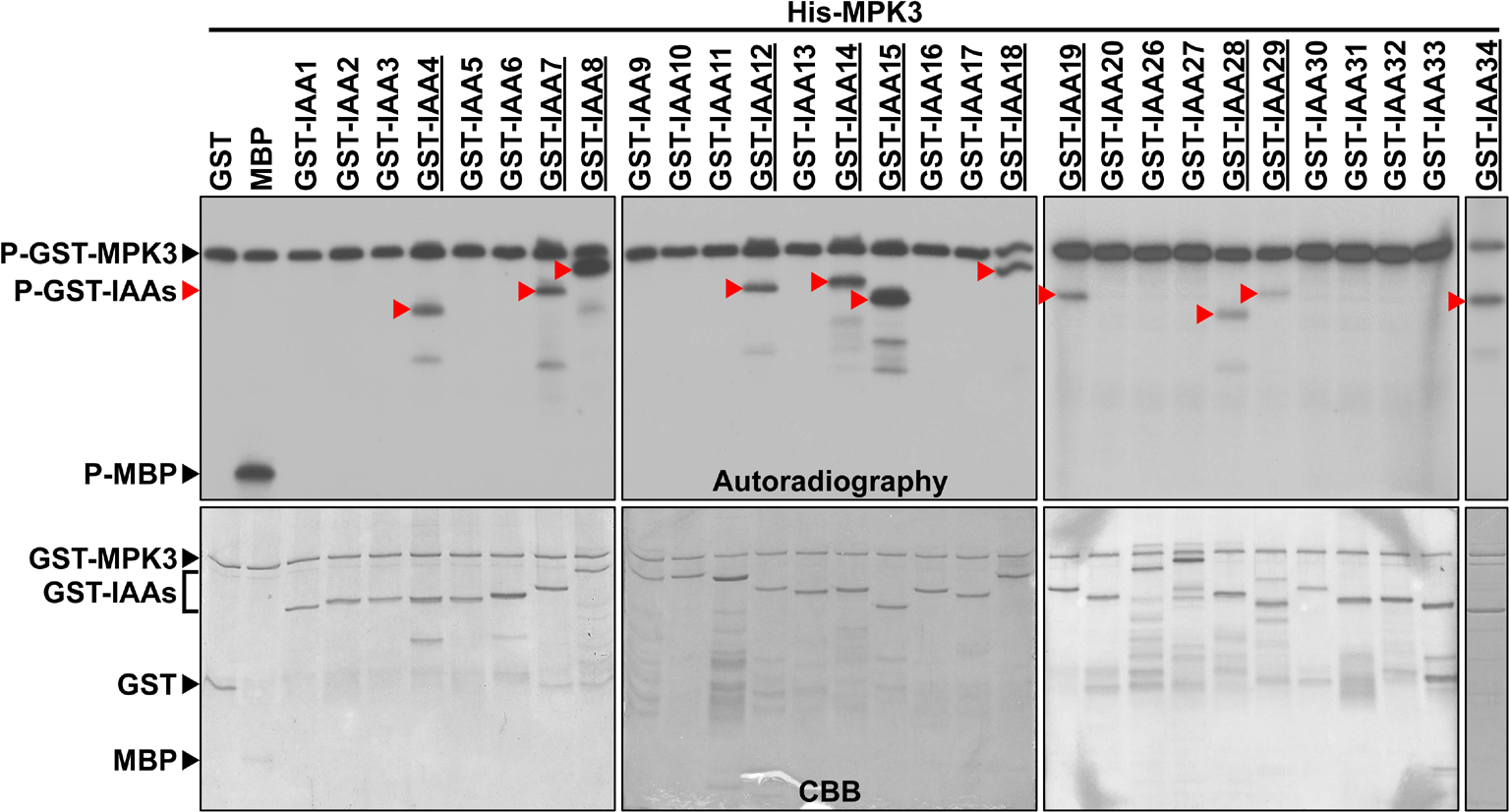


**Supplementary Figure S2.** Phosphorylation of Aux/IAA proteins by MPK3. Purified GST-fused IAA proteins were phosphorylated by an *in vitro* phosphorylation assay with GST-fused MPK3. Autoradiography (upper panel) and gels stained with Coomassie Brilliant Blue (CBB, lower panel) are shown. P-GST-MPK3 and P-MBP with black arrowheads indicate autophosphorylated MPK3 and phosphorylated MBP, respectively. Phosphorylated IAA proteins are underlined and indicated by red arrowheads. GST and MBP were used as negative and positive substrates, respectively.


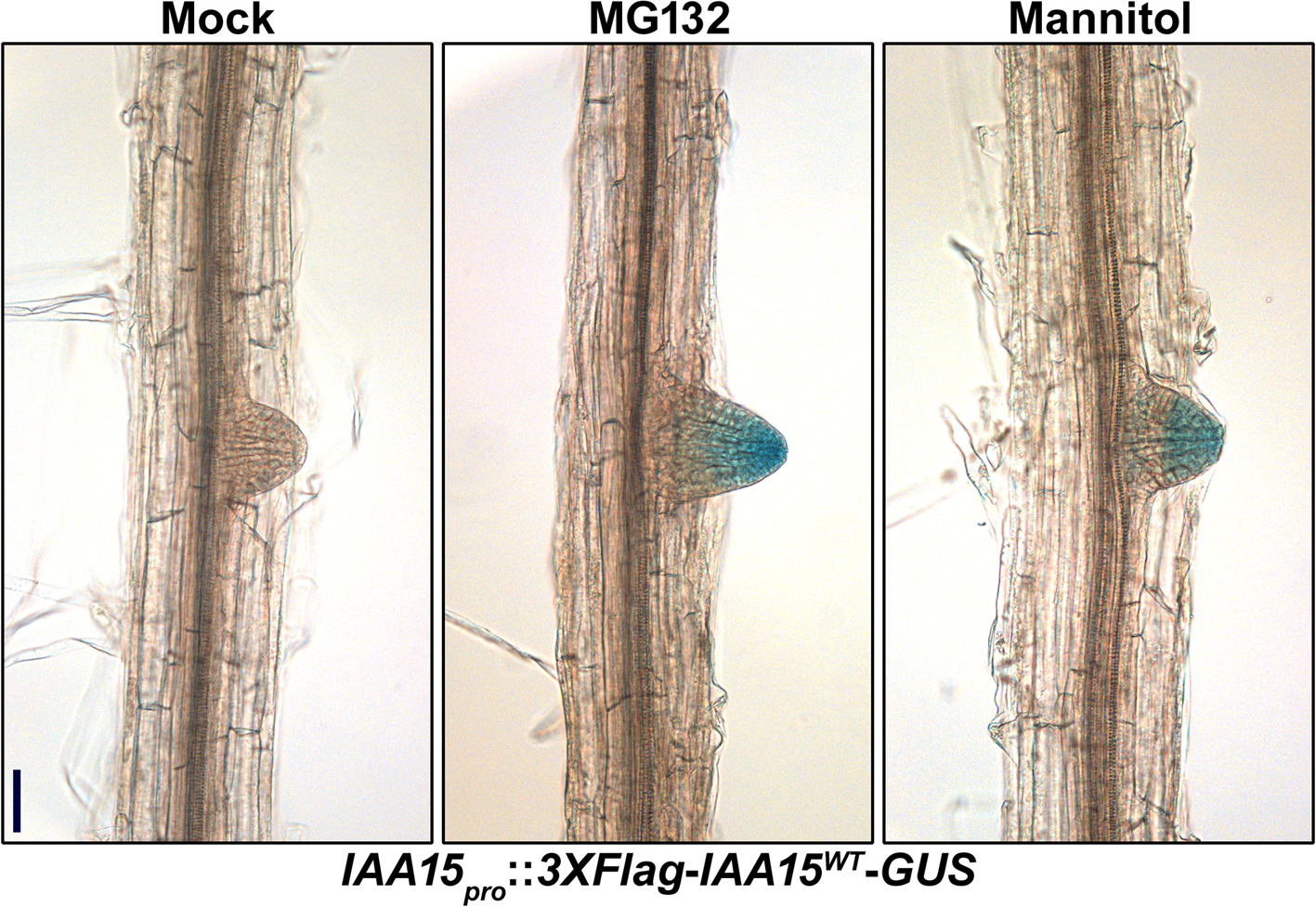


**Supplementary Figure S3.** Expression of IAA15 protein in emerged lateral roots of Arabidopsis. Ten-day-old plants of *IAA15_pro_*::*3XFlag*-*IAA15^WT^*-*GUS* transgenic plant grown vertically on MS agar plates were treated with 10 μM MG132 or 200 mM mannitol for 24 h and then were incubated for 24 h in 5-bromo-4-chloro-3-indolyl glucuronide (X-gluc) for GUS staining. The scale bars represent 50 μm.


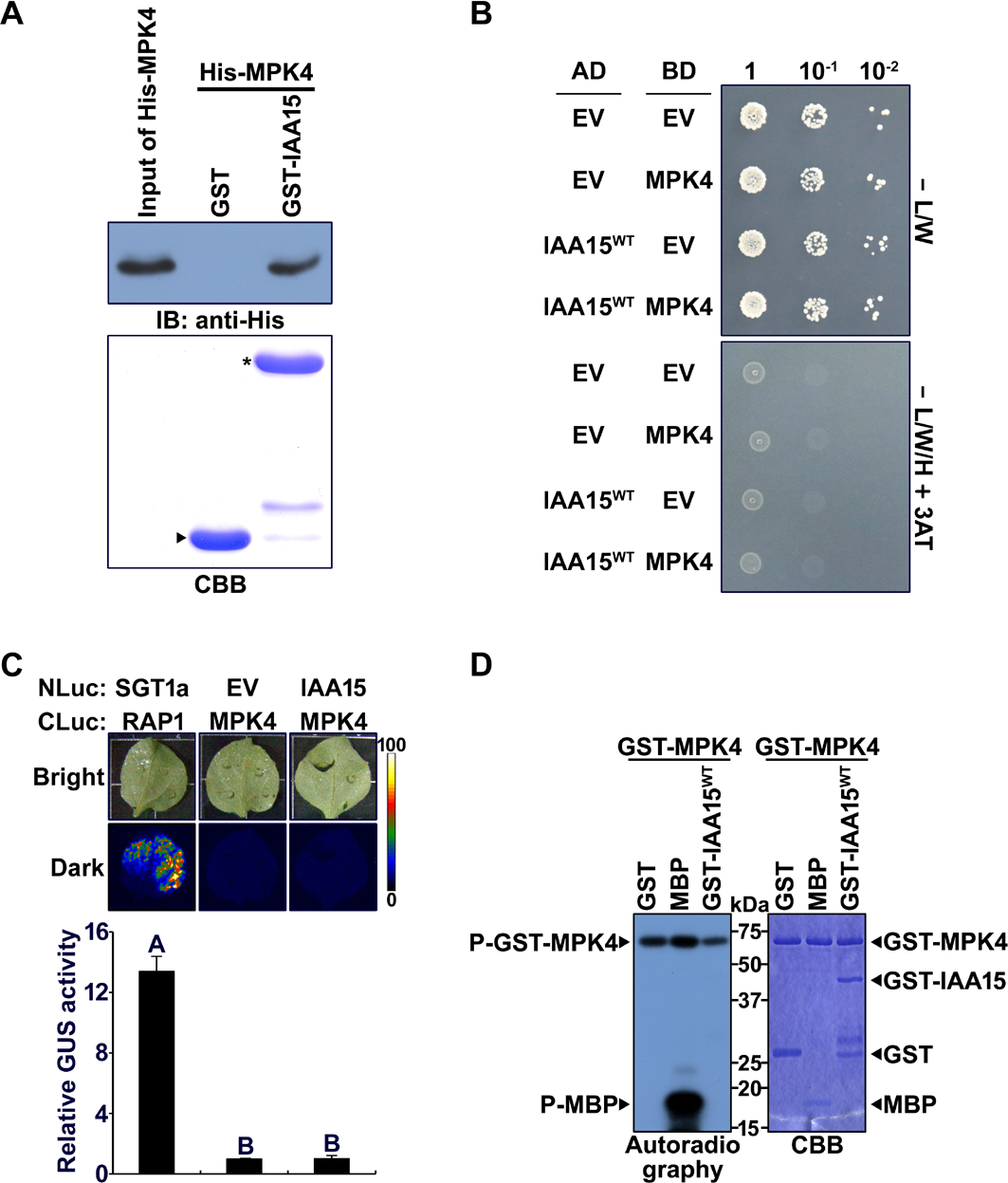


**Supplementary Figure S4.** MPK4 does not interact with and phosphorylate IAA15. **(A)** *In vitro* pull-down assay showing the interaction between IAA15 and MPK4. The interaction between GST-fused IAA15 and His-fused MPK4 was tested. GST-fused proteins were precipitated using Glutathione-Sepharose 4B resin. His-fused MPK4 were analyzed by immunoblotting using anti-His antibodies (upper panel). The SDS–PAGE gel was stained by Coomassie Brilliant Blue (CBB) staining (lower panel). Arrowheads and stars indicate GST and GST-IAA15, respectively. **(B)** Yeast two-hybrid analysis showing the interaction of IAA15 with MPK4. The indicated AD and BD plasmids were co-transformed into yeast strain pJ69-4A. Serially diluted transformants were grown on selective SD medium lacking Leu and Trp (-L/W) (control) and on SD medium lacking Leu, Trp, and His (-L/W/H) supplemented with 20 mM 3-amino-1,2,4-triazole (3-AT) to demonstrate activation of the *HIS3* reporter gene. **(C)** LCI assay showing the interaction of IAA15 with MPK4. The upper panel shows the bright field images (bright) and luminescence (dark) of *N*. *benthamiana* leaves co-infiltrated with strains containing the indicated combinations of NLuc- and CLuc-fusion constructs. The *SGT1a*-*NLuc*/*CLuc*-*RAR1* combination was used as a positive control. EV refers to empty vectors of *NLuc*. Leaves were photographed at 3 d after infiltration. The lower panel shows the quantification of LUC activity as shown in the upper panel. The luminescence intensities were measured relative to leaves infiltrated with the *NLuc* (EV)/*CLuc-MPK4* combination. The bars indicate the mean ± S.D. (*n* = 3). The bars indicate the mean ± S.D. (*n* = 3). Different letters represent significant differences determined with Student’s *t*-test (P<0.001). (**D**) *In vitro* phosphorylation of IAA15^WT^ by recombinant MPK4. Recombinant proteins were separated by 10% SDS–PAGE after incubation in protein kinase buffer containing [γ-^32^P] ATP. Phosphorylated IAA15 was detected by autoradiography after gel electrophoresis (right panel). Recombinant proteins were detected by CBB staining (left panel). MBP and GST were used as positive and negative substrates, respectively.


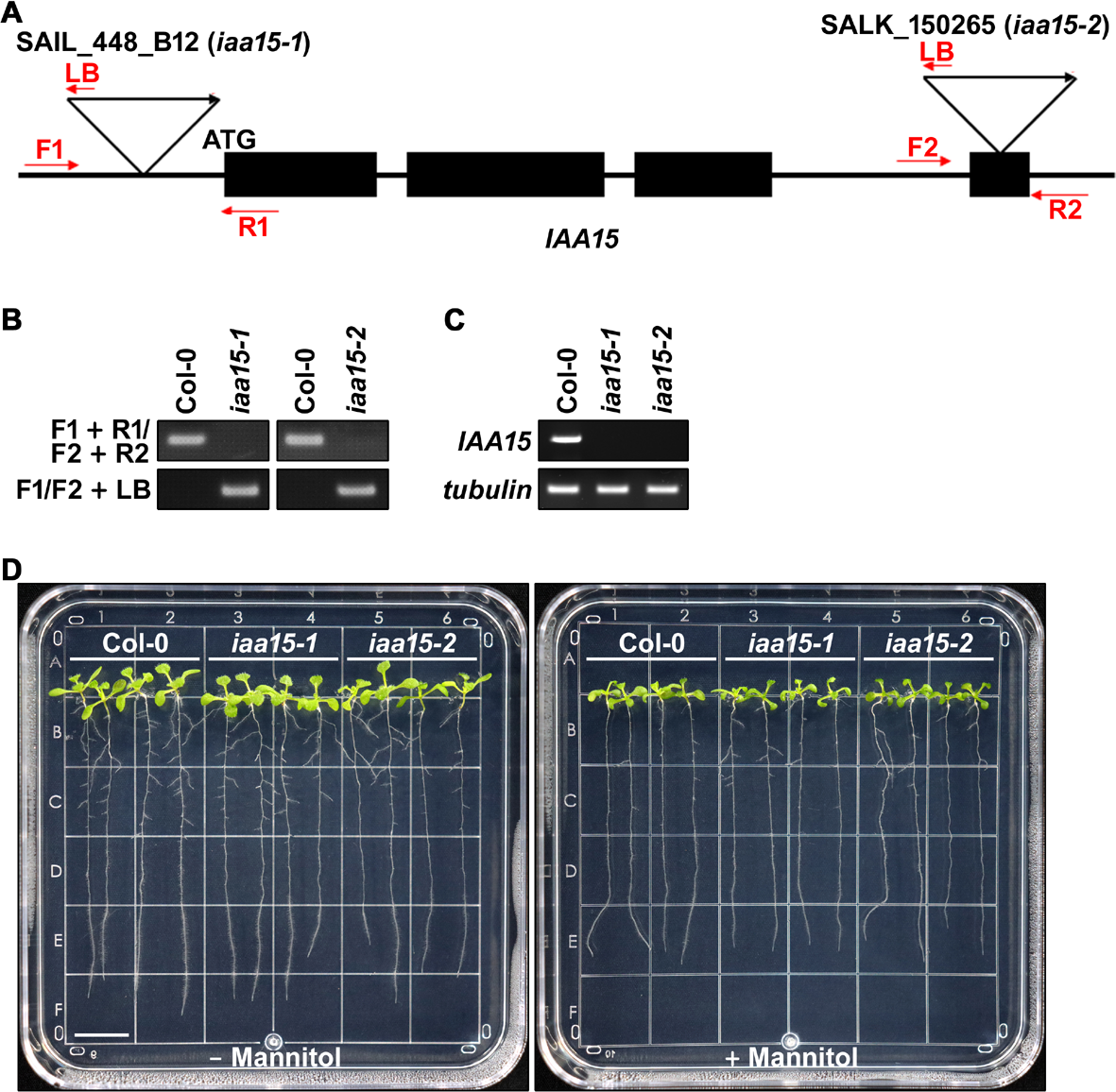


**Supplementary Figure S5.** Identification and characterization of *iaa15* loss-of-function mutants. **(A)** Schematic representation of the IAA15 gene structure and T-DNA insertion in two different *iaa15-1* (SAIL_448_B12) and *iaa15-2* (SALK_150265) mutants. **(B, C)** Analyses of genotyping **(B)** and RT-PCR **(C)** confirmed the absence of *IAA15* in homozygous *iaa15* mutants. **(D)** Root growth phenotypes of Col-0, *iaa15-1*, and *iaa15-2* plants. Shown are 11-day-old plants vertically grown on MS plates containing with or without 75 mM mannitol. The scale bar represents 1 cm.


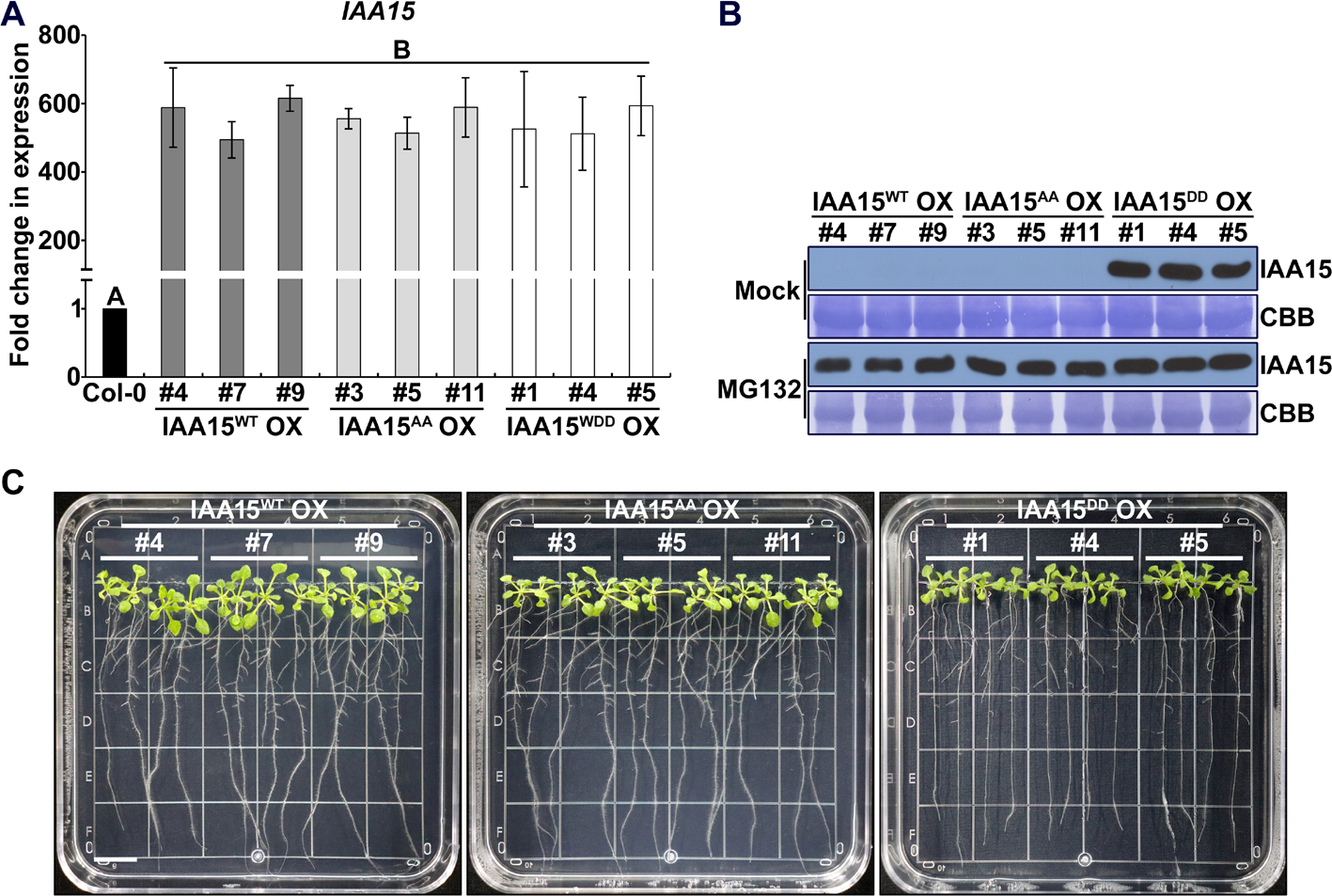


**Supplementary Figure S6.** Construction of three independent IAA15^WT^ OX, IAA15^AA^ OX, and IAA15^DD^ OX plants. **(A)** The transcript levels of *IAA15* in the IAA15 transgenic plants. Transcript levels of *IAA15* were measured by qPCR using specific primers. The bars indicate the mean ± S.D. (*n* = 3). Different letters represent significant differences (P<0.001). **(B)** Protein levels of IAA15 in IAA15^WT^ OX, IAA15^AA^ OX, and IAA15^DD^ OX plants in the absence (Mock) and presence of MG132. IAA15 proteins were detected by immunoblotting with anti-Flag antibodies. The Rubisco band stained with CBB is shown to verify similar amounts of loaded protein (lower panel). **(C)** Root phenotypes of three independent IAA15^WT^ OX, IAA15^AA^ OX, and IAA15^DD^ OX plants. Two-week-old plants grown vertically on MS agar plates were photographed. The scale bar represents 1 cm.


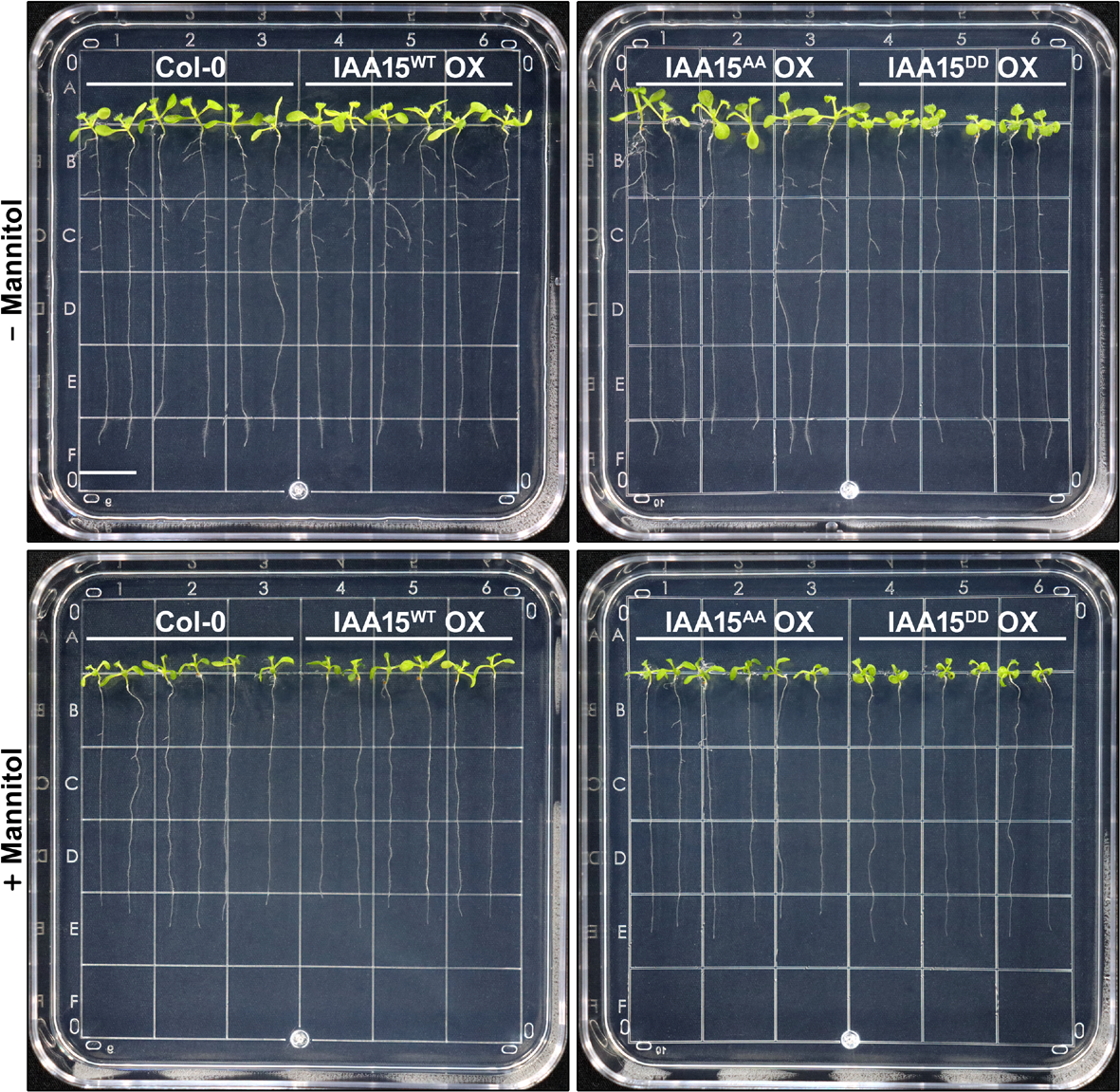


**Supplementary Figure S7.** Root phenotype of Col-0, IAA15^WT^ OX #7, IAA15^AA^ OX #5, and IAA15^DD^ OX #1 plants in response to drought. Four-day-old plants grown on MS medium were transferred on MS plates containing with or without 75 mM mannitol and grown vertically for 7 d. The scale bar represents 1 cm.


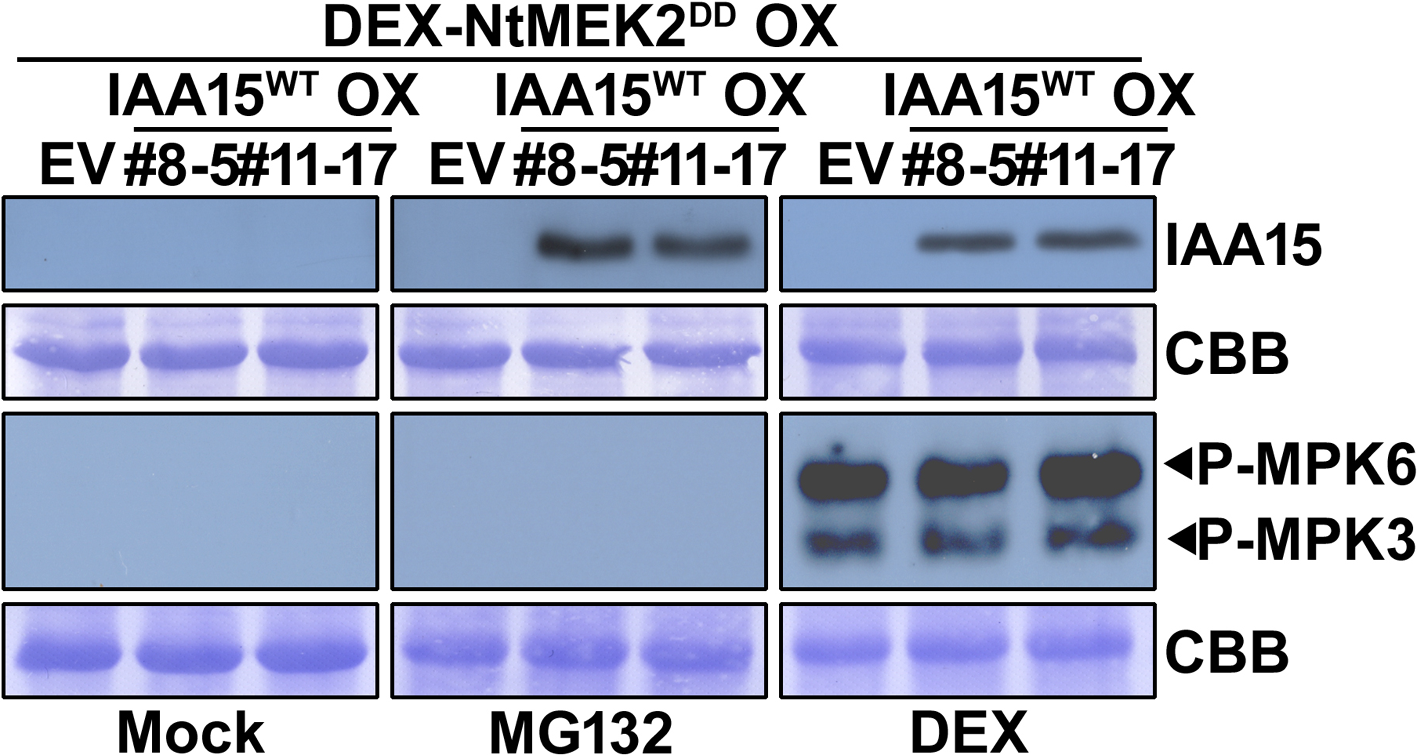


**Supplementary Figure S8.** Stabilization of IAA15 requires the activation of MPK3 and MPK6. Immunoblotting analyses were performed in double transgenic plants overexpressing HA-fused IAA15^WT^ and Flag-fused NtMEK2^DD^. The plants were treated with mock, MG132, and DEX treatments. MPK3 and MPK6 activities and IAA15 proteins were detected by immunoblotting with anti-p44/42 and anti-HA antibodies, respectively. The Rubisco band stained by CBB is shown to verify similar amounts of loaded protein (lower panel).


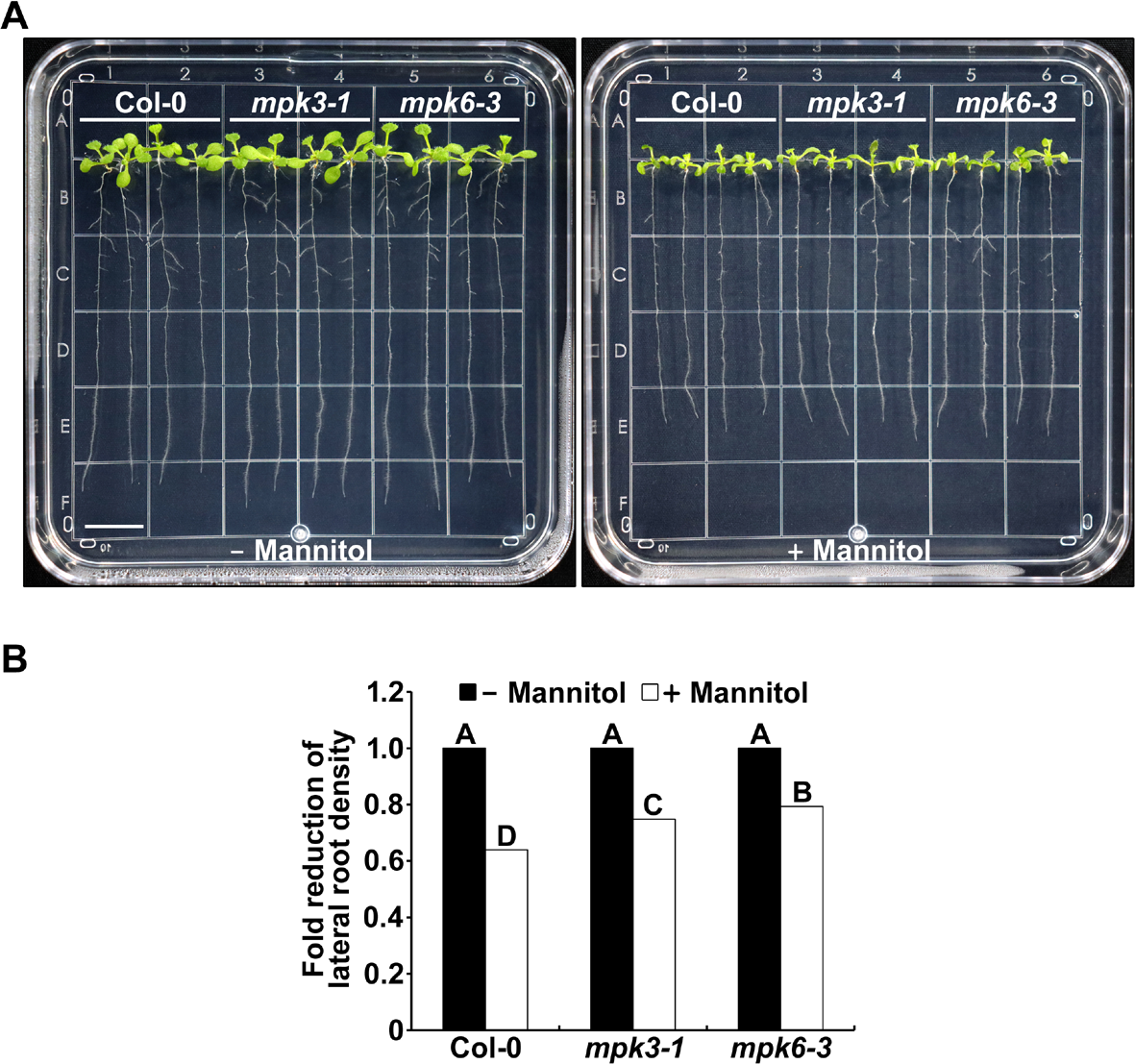


**Supplementary Figure S9.** Root phenotype of Col-0, *mpk3-1, and mpk6-3* plants in response to drought. (A) Four-day-old plants grown on MS medium were transferred on MS plates containing with or without 75 mM mannitol and grown vertically for 7 d. The scale bar represents 1 cm. (B) Fold reduction of lateral root density in Figure 5F. Different letters represent statistically significant differences between genotypes (*P* < 0.05).


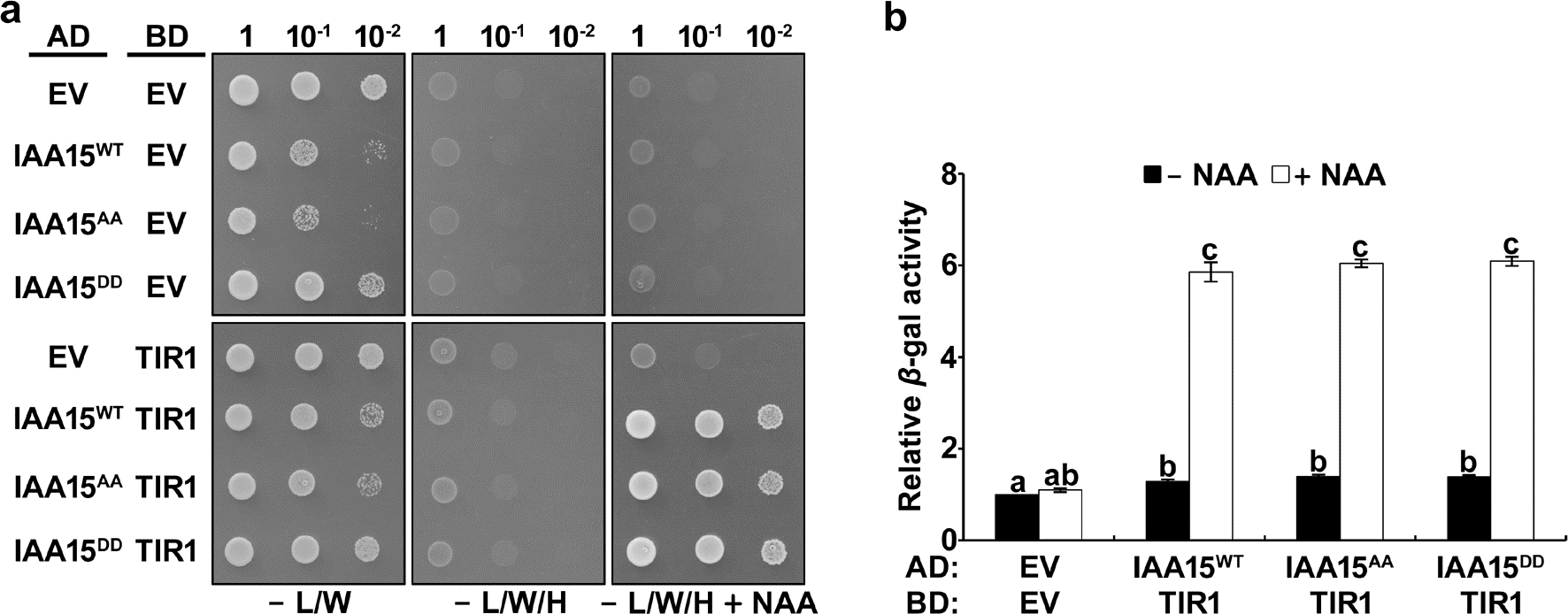


**Supplementary Figure S10.** Yeast two-hybrid analysis showing the interaction between IAA15^WT^ or its mutants and TIR1. **(A)** The indicated AD and BD plasmids were co-transformed into the yeast strain pJ69-4A. Serially diluted transformants were grown on selective SD medium lacking Leu and Trp (-L/W) (control) and on SD medium lacking Leu, Trp, and His (-L/W/H) in the absence and presence of NAA. **(B)** *β*-galactosidase (*β*-gal) assay showing the interaction of IAA15^WT^ and its mutants with TIR1 in the absence and presence of NAA. The *β*-gal activity was analyzed in liquid cultures of yeast cells using ortho-nitrophenyl-*β*-galactoside (ONPG) as a substrate. The bars indicate the mean ± S.D. (*n* = 3). Different letters represent statistically significant differences between genotypes and IAA treatments (*P* < 0.05).


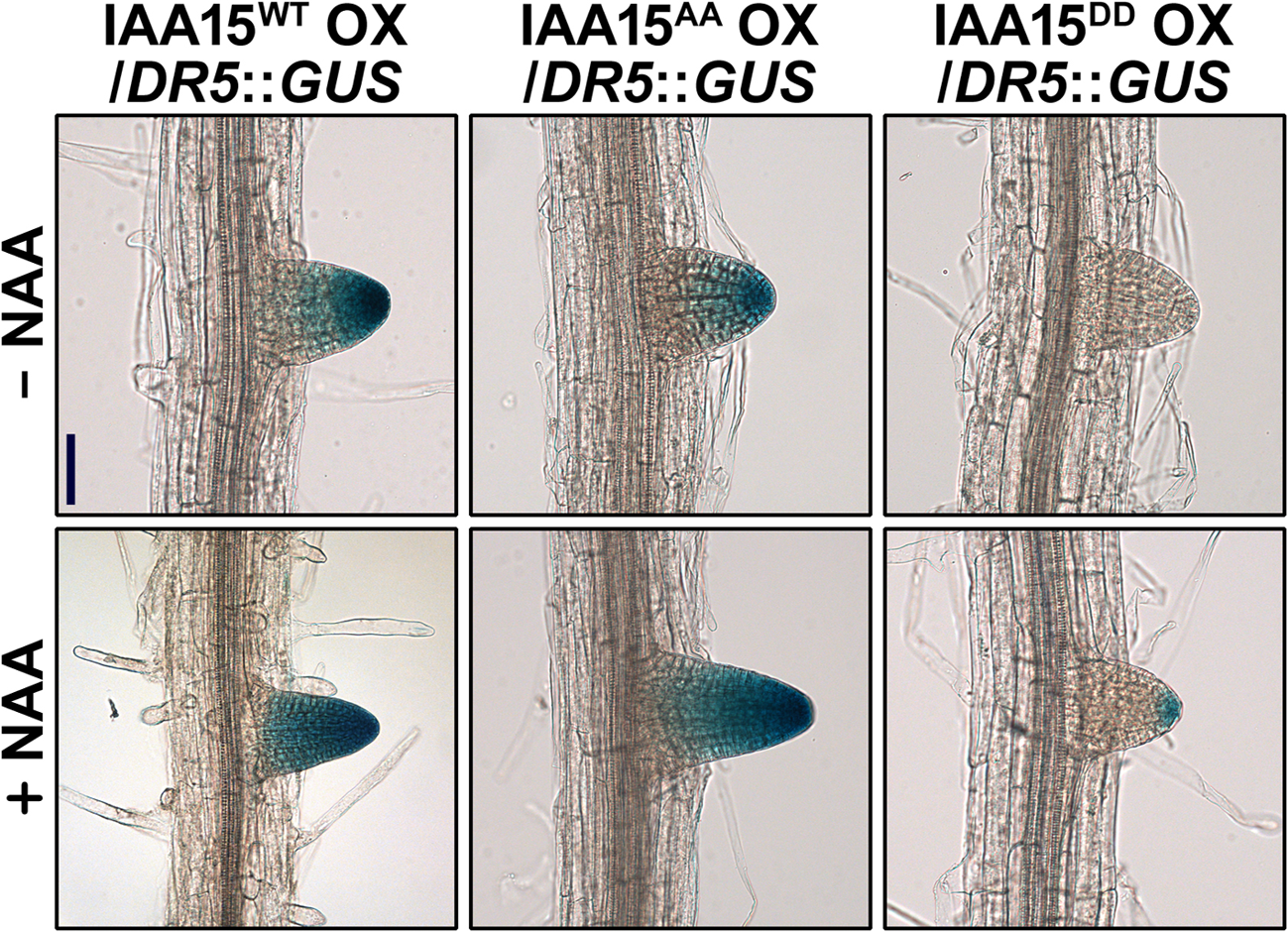


**Supplementary Figure S11.** Inhibition of the auxin-responsive *DR5*::*GUS* reporter in IAA15^DD^ OX plants. Seven-day-old plants of IAA15^WT^ OX/*DR5*::*GUS*, IAA15^AA^ OX/*DR5*::*GUS*, and IAA15^DD^ OX/*DR5*::*GUS* vertically grown on MS plates were treated with or without NAA for 12 h. These plants were incubated for 12 h in 5-bromo-4-chloro-3-indolyl glucuronide (X-gluc) for GUS staining, and the emerged lateral root were photographed. The scale bar represents 50 μm.


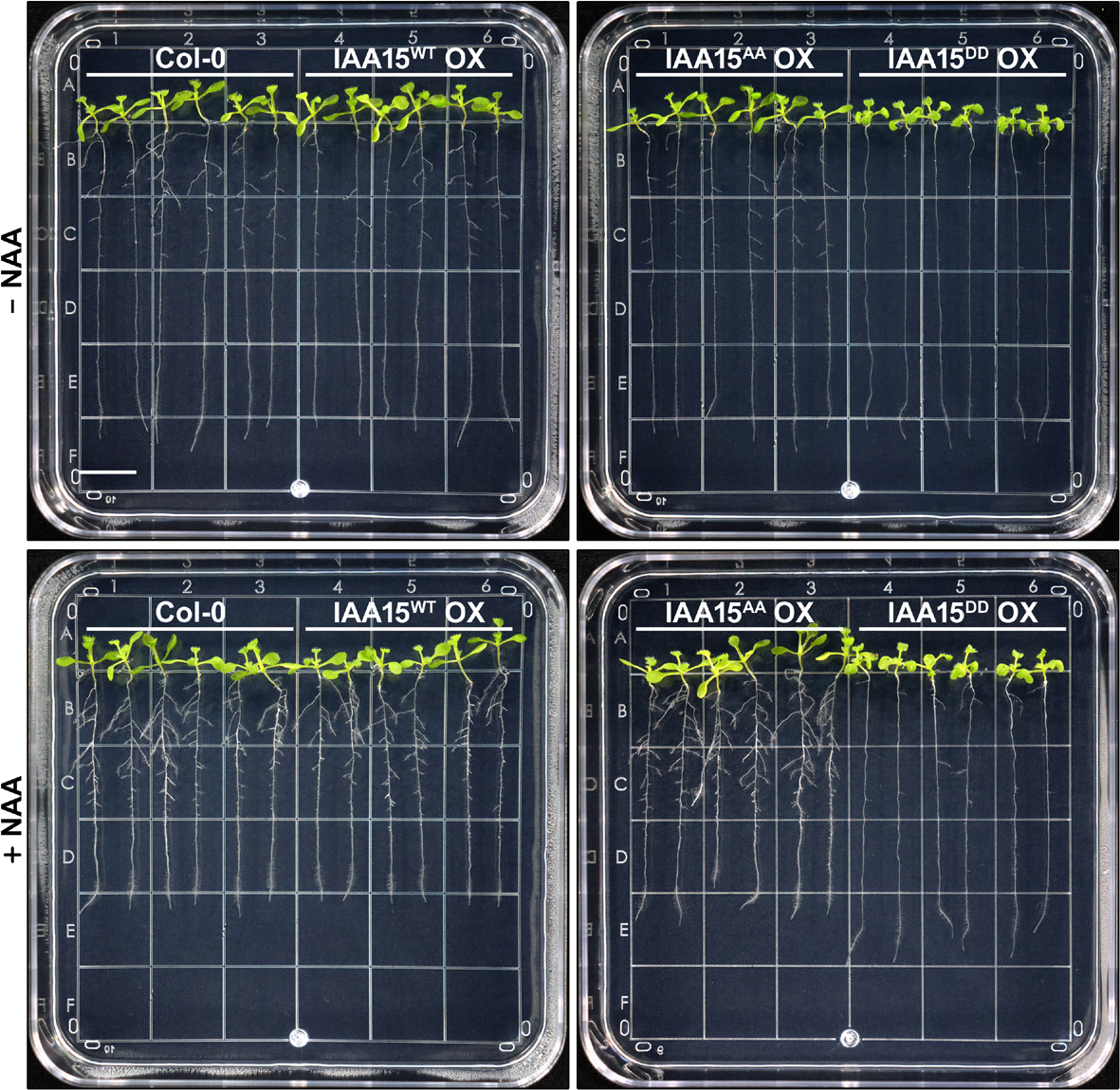


**Supplementary Figure S12.** Root phenotype of Col-0, IAA15^WT^ OX #7, IAA15^AA^ OX #5, and IAA15^DD^ OX #1 plants in response to auxin. Four-day-old plants grown on MS medium were transferred on MS plates containing with or without 25 nM NAA and grown vertically for 7 d. The scale bar represents 1 cm.


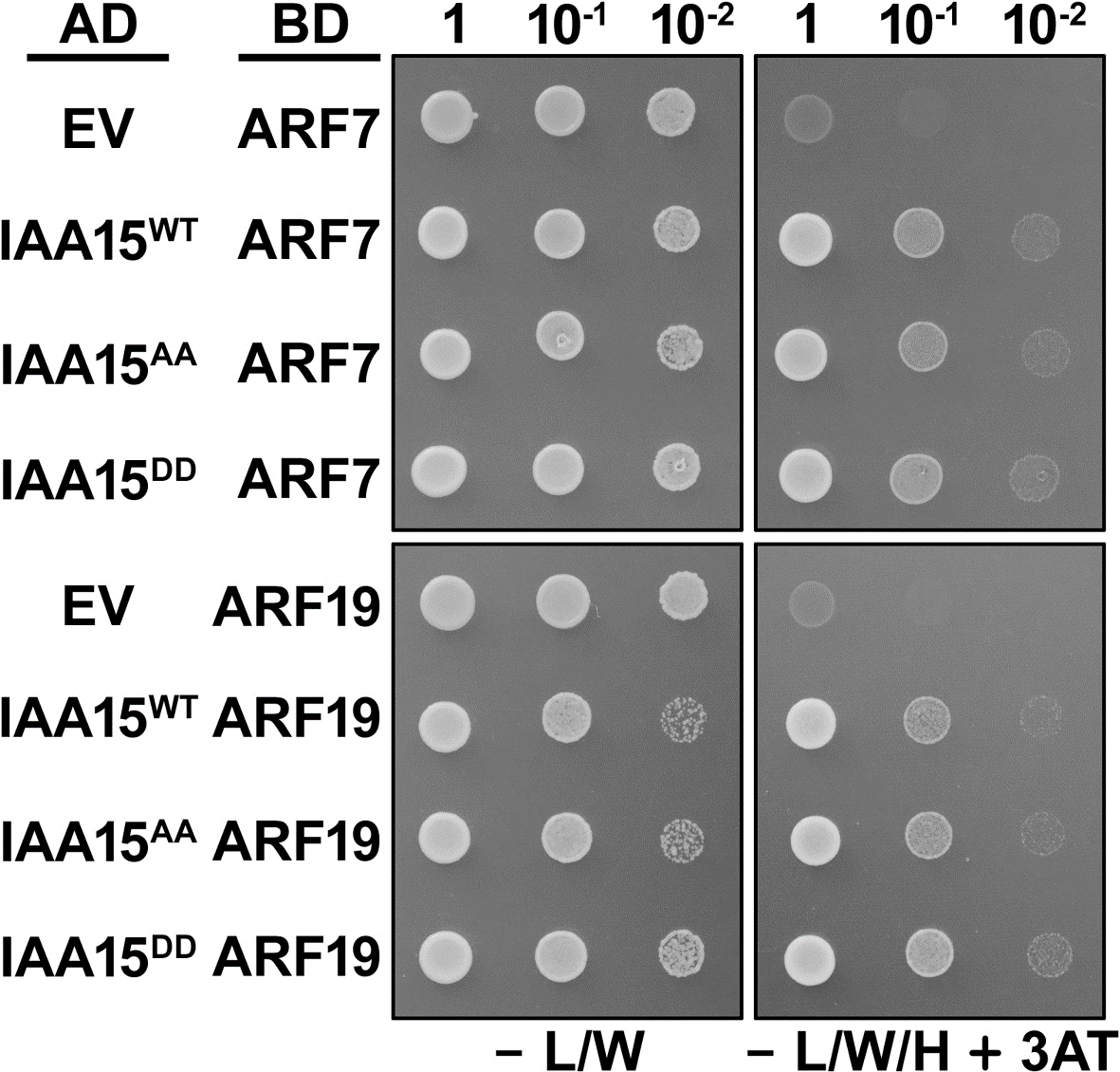


**Supplementary Figure S13.** Yeast two-hybrid assay showing the interaction between IAA15^WT^ or its mutants and ARF7 or ARF19. The indicated AD and BD plasmids were co-transformed into yeast strain pJ69-4A. Serially diluted transformants were grown on selective SD medium lacking Leu and Trp (-L/W) (control) and on SD medium lacking Leu, Trp, and His (-L/W/H) supplemented with 20 mM 3-amino-1,-2,-4-triazole (3-AT) to demonstrate activation of the *HIS3* reporter gene.


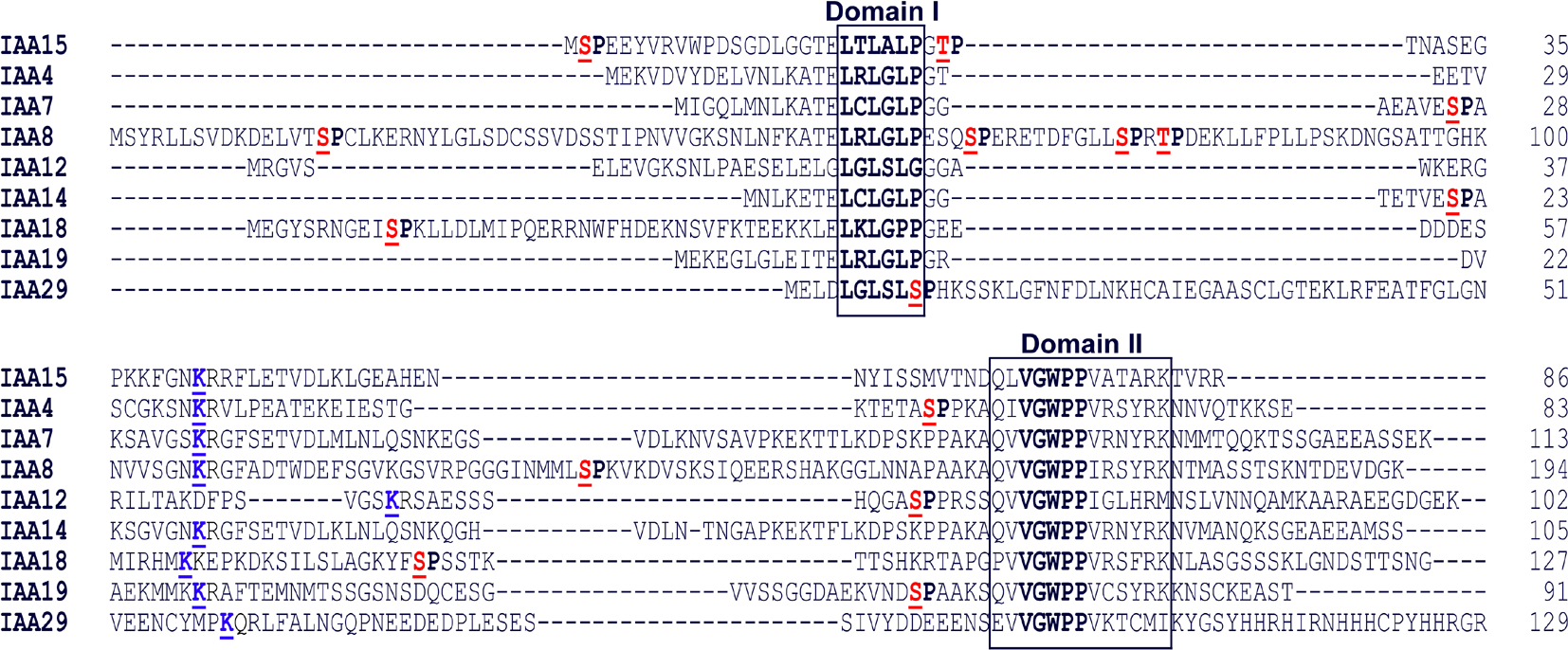
 **Supplementary Figure S14.** Alignment of 9 canonical Aux/IAA proteins as putative substrates of MPK3. The N-terminal amino acids (including domain I and domain II) of 9 Aux/IAAs phosphorylated by MPK3 (in shown Supplementary Figure S2) were aligned using the ClustalW program (version 2.1, https://www.genome.jp/tools-bin/clustalw). Conserved domains I and II were boxed. Docking motif (VGWPP) of the SCF^TIR1/AFB^ complex in domain II is bold letters. Underlined red (S and T) and blue (K) color letters indicate the predicted phosphorylation and ubiquitination sites, respectively.


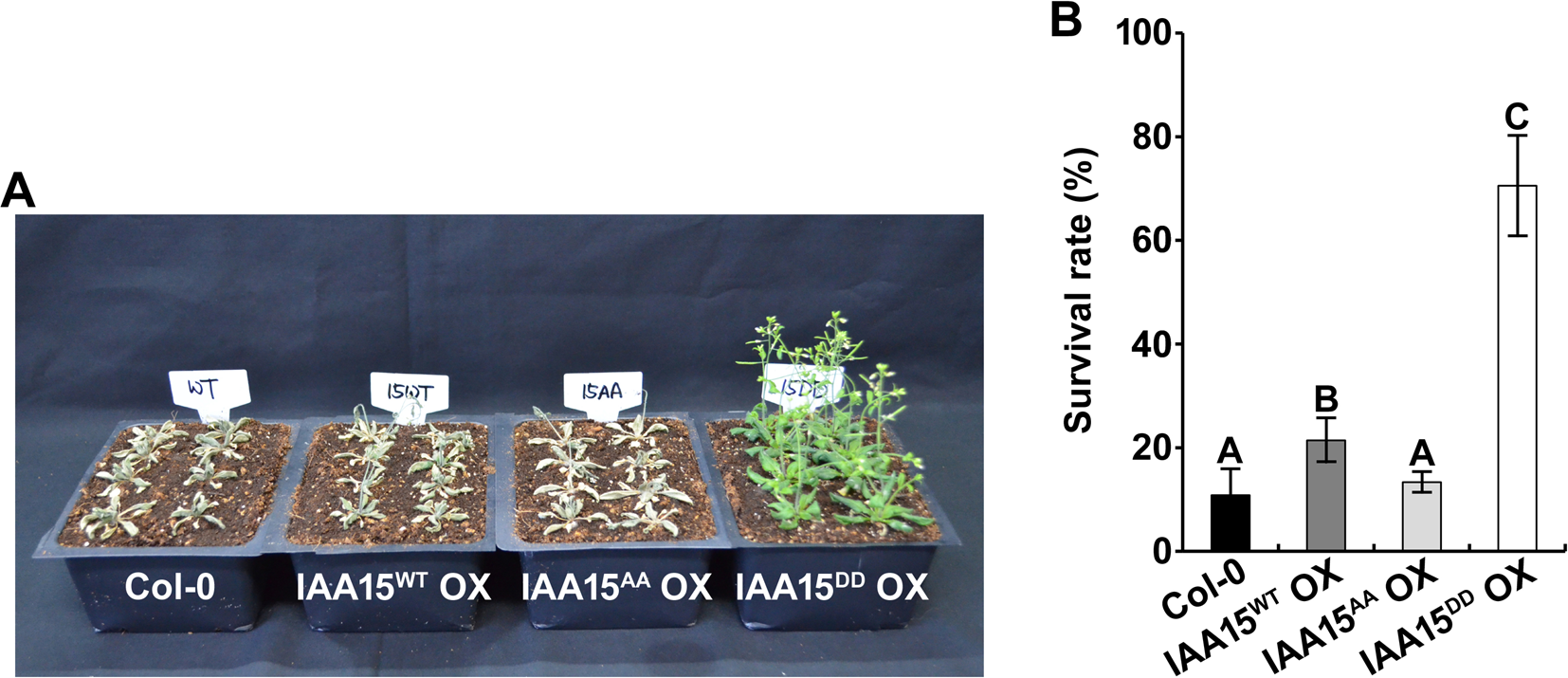


**Supplementary Figure S15.** IAA15^DD^ OX plants show enhanced drought tolerance. **(A)** Drought tolerance of IAA15^DD^ OX plants. Two-week-old plants were not watered for 2 weeks and photographed after re-watering for 5 d. **(B)** Increased survival rate of IAA15^DD^ OX plants. At least 36 plants for each individual line were used to evalutate suvival rate. The bars indicate the mean ± S.D. (*n* = 3). Different letters represent statistically significant differences between genotypes (*P* < 0.001).

**Supplemental Table S1.** Primers used for cloning and site-directed mutagenesis.

| **Construct** | **Position** | **Sequence** |
| --- | --- | --- |
| *IAA15^WT^* | F | 5’- *ggatccATGTCACCGGAGGAATACGT* -3’ |
|  | R | 5’- *actagtTCACTTACATATTGTTATTA* -3’ |
| *IAA15^S2A^* | F | 5’- *GGATCCATGGCACCGGAGGAATACGTTAGG* -3’ |
|  | R | 5’- *TTCCTCCGGTGCCATGGATCCACGCGGAAC* -3’ |
| *IAA15^T28A^* | F | 5’- *TTGCCTGGCGCTCCGACAAATGCATCGGAA* -3’ |
|  | R | 5’- *ATTTGTCGGAGCGCCAGGCAAAGCTAGGGT* -3’ |
| *MPK3* | F | 5’- *ggatccATGAACACCGGCGGTGGCCA* -3’ |
|  | R | 5’- *gtcgacCTAACCGTATGTTGGATTGA* -3’ |
| *MPK4* | F | 5’- *ggatccATGTCGGCGGAGAGTTGTTT* -3’ |
|  | R | 5’- *gtcgac*ATGTCGGCGGAGAGTTGT*AT* -3’ |
| *MPK6* | F | 5’-ggatccATGGACGGTGGTTCAGGTCA -3’ |
|  | R | 5’-gtcgacCTATTGCTGATATTCTGGAT -3’ |
| *IAA15*  (For gateway system) | F | 5’- *aaaaaagcaggcttaATGTCACCGGAGGAATACGT* -3’ |
|  | R | 5’- agaaagctgggtcTCACTTACATATTGTTATTA -3’ |
| *ARF7*  (For gateway system) | F | 5’- aaaaaagcaggcttaATGAAAGCTCCTTCATCA -3’ |
|  | R | 5’- agaaagctgggtcTCACCGGTTAAACGAAGT -3’ |
| *ARF19*  (For gateway system) | F | 5’- aaaaaagcaggcttaATGAAAGCTCCATCAAAT -3’ |
|  | R | 5’- agaaagctgggtcCTATCTGTTGAAAGAAGC -3’ |
| *TIR1*  (For gateway system) | F | 5’- aaaaaagcaggcttaATGCAGAAGCGAATAGCC -3’ |
|  | R | 5’- agaaagctgggtcTTATAATCCGTTAGTAGT -3’ |
| *MPK3*  (For gateway system) | F | 5’- aaaaaagcaggcttaATGAACACCGGCGGTGG -3’ |
|  | R | 5’- agaaagctgggtcCTAACCGTATGTTGGATT -3’ |
| *MPK4*  (For gateway system) | F | 5’- aaaaaagcaggcttaATGTCGGCGGAGAGTTGT -3’ |
|  | R | 5’- agaaagctgggtcATGTCGGCGGAGAGTTGT -3’ |
| *MPK6*  (For gateway system) | F | 5’- aaaaaagcaggcttaATGGACGGTGGTTCAGGT -3’ |
|  | R | 5’- gtcgacCTATTGCTGATATTCTGGAT -3’ |

**Supplemental Table S2.** Primers used for qPCR and ChIP‒qPCR.

| **Construct** | **Position** | **Sequence** |
| --- | --- | --- |
| *IAA15* | F | 5'- AACGTAGATTCCTCGAGACC -3' |
|  | R | 5'- TCTAGAGCGGTGAAAAGCTG -3' |
| *LBD16* | F | 5'- GAGAGACTCATCATCAAACC -3' |
|  | R | 5'- CTAAGAGCCAAAGCCTGAAG -3' |
| *LBD29* | F | 5'- AAGTTCTGGGACGGTTCAAC -3' |
|  | R | 5'- GCTGATTGAAGCTCTTTGAG -3' |
| *LBD16 promoter P1* | F | 5'- AACCCAATAAATTAGAAGTC -3' |
|  | R | 5'- CAAAGGGTGTGAATGGTAGA -3' |
| *LBD29 promoter P1* | F | 5'- CCTACGTACTGTTCAAATAT -3' |
|  | R | 5'- CTTTAACTTATATTTCTAAC -3' |
| *LBD29 promoter P2* | F | 5'- TTCCACAACTGCTATGCGAT -3' |
|  | R | 5'- TGATATTTTCGTATCATTTA -3' |
| *Tubulin* | F | 5'- CCAACAACGTGAAATCGACAG -3' |
|  | R | 5'- TCTTGGTATTGCTGGTACTCT -3' |

**Supplemental Table S3.** Identification of phospho-peptides by mass spectrometry analysis via TiO_2_ chromatography from the tryptic digestion of IAA15 phosphorylated by MPK6.

| **Phospho-peptide sequence** | **Position of IAA15** | **No. of**  **phosphate groups** | **(M+H)** | | | **Putative phosphorylation site** |
| --- | --- | --- | --- | --- | --- | --- |
|  |  |  | **Expected** | | **Measured** |  |
| GSMS^*^PEEYVR | -2 to 8 | 1 | 1234.48 | 1234.42 | | Ser-2 |
| VWPDSGDLGGTELTLALPGT^*^PTNASEGPK | 9 to 37 | 1 | 2960.39 | 2960.41 | | Thr-28 |

^*^Amino acid residues that could potentially be phosphorylated by MPK6

**Supplemental Table S4.** Characteristics of a gain-of-function mutant (IAA15^P78S^ OX) (25) and phospho-mimicking mutant (IAA15^DD^ OX) of IAA15.

| **Characteristics** | **IAA15^WT^ OX** | **IAA15^DD^ OX** | **IAA15^P78S^ OX** |
| --- | --- | --- | --- |
| ***In planta* polyubiquitination** | High | Low | Very low |
| **Protein stability^1^** | Unstable^a^ | Stable^b^ | Very stable^c^ |
| **Relative lateral root growth** | 100% | 54% | 10% |

^1^Protein stabilities of three different IAA15 proteins were measured by *in vivo* turnover assay.

^a^Approximately 2% of IAA15^WT^ protein was detected at 4 h after the transfer to media containing CHX (25; Figure 6B).

^b^Approximately 42% of IAA15^DD^ protein was detected at 4 h after the transfer to media containing CHX (Figure 6C).

^c^Approximately 80% of IAA15^P78S^ protein was detected at 4 h after the transfer to media containing CHX (25).
